# The cherry on top: nice but necessary? The impact of social support on perceived stress, depressive symptoms, and academic performance among medical students at Damascus university

**DOI:** 10.1186/s40359-025-03329-0

**Published:** 2025-09-30

**Authors:** Amr Hadaki, Ahmad Snowbar, Ahmad Alsayes, Alma Shoqeir, Tala Musa, Muhammed Besher Manlarasool, Youssef Latifeh

**Affiliations:** 1 Third-Year Medical Student, Faculty of Medicine, Damascus University, Damascus, Syria; 2 Department of Psychiatry, Faculty of Medicine, Damascus University, Damascus, Syria

**Keywords:** Social support, Stress, Depression, Academic performance, Medical students

## Abstract

**Background:**

Students often face many struggles with their university career especially the large amount of stress which is considered an important factor affecting them. Many students consider social support to be a great factor to overcome this stress. This study aims to measure the level of stress and depressive symptoms among medical students at Damascus University and the social support they received to examine the relationship between these variables on the academic performance of the students.

**Purpose:**

To investigate the association between social support received by medical college students and their academic performance and mental wellbeing at Damascus University.

**Method of study:**

A cross-sectional study was conducted using an online self-administered questionnaire. An appropriate sample representing all students of medical colleges at Damascus University was randomly selected to complete the questionnaire.

**Results:**

A total of (1217) medical students participated in the study. Among them, (65.7%) were females and (34.3%) were males. Social support had no correlation with grades of the last semester when spearman’s test was conducted nor with perceived stress. Female students received more high and moderate social support (High social support revenue using MSPSS scale: Females: 59%, Males: 49%; P-value = 0.002) and had higher rates of depressive and anxiety symptoms as shown by using PHQ4 scale (moderate depressive and anxiety symptoms: Females = 46%, Males = 35%; P-values < 0.001). Anxiety was correlated with the faculty of students and with the score of the last semester. The majority of participants experienced moderate levels of stress (*n* = 630, 51.8%). Academic-related stressors were the most prevalent cause of stress among students, especially female students.

**Conclusion:**

Our results indicated no significant relationship between social support or perceived stress and the academic performance of the students. One possible explanation for this finding could be that students are more self-dependent and self-reliant. Female medical students suffered higher levels of stress, particularly academic-related stress, compared to their male peers. Students who received high levels of social support expressed less depressive and anxiety symptoms compared to those who received less social support.

**Supplementary Information:**

The online version contains supplementary material available at 10.1186/s40359-025-03329-0.

## Introduction

The transition from high school to college presents students with a myriad of new challenges that can significantly impact their psychological well-being, particularly for those who choose to enroll in medical colleges [1], These students are often confronted with a fast-paced and demanding curriculum, coupled with a competitive atmosphere that characterizes many medical institutions [2]. As a result, they frequently experience what is known as “imposter syndrome” [3], which can adversely affect their daily lives and overall health. Research indicates that medical students report higher rates of depression [4], anxiety [5], burnout [6], substance misuse [7] and suicidal ideation [4] compared to their age-matched peers in the general population [8].

In the past few decades, perceived social support drew significant attention among researchers from all around the world (Us [8–10], Turkey [11], Brazil [12], South Korea [13], China [14], Germany [15]). Which is an obvious affordable way for students to deal with such educational stress [9] which is known as assisting and comforting other people in order for them to be able to cope with a variety of problems. Such assistance can come from within the family or from personal relations such as neighbors, religious groups, or friends in general [16]. Thus, social support can be defined as “The existence or availability of people on whom we can truly rely, people who let us know that they care about, value and love us” [17]. When asked to address who they turn to in times of crisis and emotional distress, people typically mention their family members and friends, their “natural helpers” [18]. Exchanging personal, social, or moral ideas between peers supports each individual and his/her social development [16].

A The prevalence of anxiety and depression among medical students has been documented worldwide [19, 20]. Recent studies suggest that medical schools may foster a toxic psychological environment [21, 22], exacerbated by factors such as immense workloads, financial pressures, and sleep deprivation, all of which contribute to elevated stress and anxiety levels. Symptoms of depression are often characterized by feelings of hopelessness, sadness, and anxiety [23]. These manifestations can significantly impair medical students, leading to declines in academic performance, increased dropout rates, substance abuse, and even suicide [24–27].

Previous research has underscored the positive impact of social support on adolescents’ adjustment, regardless of the stress levels they encounter [28]. One study reported results that indicated a direct significant relationship between social support and academic success [29]. Another study highlighted a negative correlation between the stress experienced by college students and the social support they received [11]. However, some studies found no significant effects of either receiving or providing social support on global cognition and recognition [30]. Notably, a significant moderation effect was observed for memory; those who received social support exhibited stronger associations with higher working memory among less-educated individuals compared to their more educated counterparts [30].

However, some conducted studies suggested the existence of limiting factors that can affect the correlation between social support and stress. Some of these studies [10] observed that college students who perceived less social support expressed more depressive symptoms, but when symptoms of depression were controlled, social support did not predict perceived stress. Another study [31] on African-American college students also showed that social support did not predict perceived stress and furthermore, it did not have a moderator function on the relationship between racial discrimination and perceived stress. In another study [32], the negative relation between emotional support and perceived stress in college students lost its significance when focusing on problem solving and coping with stress was fully mediated. Consequently, that study concluded that the quality of the relationship between social support and perceived stress may vary based on individuals’ positive or negative experiences [11].

No previous research that studied perceived social support has been conducted in Syria, nevertheless one study in particular pertained to the Syrian population [33]. However, it mainly focused on stress levels without sufficient information on the social support aspect of medical students. Since academic stress is often associated with detrimental effects on students’ academic performance [34], and considering that Syria has recently emerged from a devastating war lasting over 14 years— which severely affected the country’s infrastructure, education system, and the psychological well-being of its students [35]- this study aims to explore the relationship between the perceived social support students receive and their academic performance and psychological stability. The goal is to enhance mental well-being and foster development during this critical juncture in the country’s history.

### Main hypothesis

There is a positive association between social support and students’ academic performance.

## Methodology

### Participants and procedure

The participants of this study were a total of 1310 students from the three medical faculties (Medicine, Dentistry and Pharmacy), who voluntarily agreed to participate. Students were informed about the purpose of the study and were not compensated for their participation. 94 responses (7.1%) were omitted due to incomplete or inappropriate responses (illogical or missing answers in the demographic section); these responses however didn’t affect the general demographics of the sample and did not show any significant demographic patterns.

An online, self-administered questionnaire was issued to the students, between April and July of the year 2024. No personal data was gathered, and participants were assured of the anonymity of their responses.

Inclusion Criteria: the study will include students enrolled at Damascus University from any of the following three faculties: Medicine, Pharmacology, and Dentistry. Eligible participants must be in their second to sixth academic year.

Exclusion Criteria: students with diagnosed mental health issues and those who already have graduated will be excluded from this study.

To mitigate response bias that occurs in online surveys, we assured the participants the anonymity of the responses to reduce the social desirability bias which is a form of response bias where participants tend to give answers in a way that they believe is more socially acceptable or favorable. Additionally, a simple random sampling method was employed when contacting the students to ensure that the sample was representative of the broader population (randomizing the students list and contacting students from the top of the randomized list). This study was approved by the Biomedical Research Ethics Committee of Damascus University (ID number: MD-020624-249).

### Measures

#### Demographics

General characteristics of the population were obtained including gender, age, whether any of the parents is a doctor, educational level of the parents, social status, income source, grade of last semester and economical status.

#### Stress: medical student stress scale (MSSQ)

A 40-item scale was constructed by Yusoff et al. [36, 37], to identify sources of stress for medical students and the intensity of stress caused by each one of them. The questionnaire manual divided the questions into six domains based on a common theme. It is considered a Likert-type questionnaire where students can answer each question with one of the 5 answers ‘causing no stress at all’, ‘causing mild stress, ‘causing moderate stress’, ‘causing high stress’ and ‘causing severe stress’. This scale was chosen to evaluate the amount of stress that students are exposed to and determine the sources of it. The scale was validated and translated into Arabic [36, 38].

The MSSQ is a valid scale with high reliability that evaluates the amount and sources of stressors that medical students are exposed to so we found it to be a suitable scale to be used in our study.

#### Social support: multidimensional scale of perceived social support (MSPSS)

We have chosen the Arabic translation of MSPSS scale to measure social support for the students. It’s a 12-item scale developed by Zimet, Dalhem, Zimet, & Farley [39] and translated by Rana Merhi and Shahe S. Kazarian [40], which measures the amount of social support students receive from different sources. Each item is rated on a seven-point Likert-type response format (1 = very strongly disagree; 7 = very strongly agree). The total score is calculated as the mean of all of the items. The higher the score is, the more social support the student perceives. Furthermore, three independent subscales can be constructed separating the questions into three groups (Significant other, family, and friends).

Several peer-reviewed articles have validated this scale to have good psychometric properties [41, 42] along with other articles validating the translated version [40, 43].

#### Depression and anxiety: the Four-Item patient health questionnaire for anxiety and depression (PHQ-4)

The Patient Health Questionnaire (PHQ-4), developed and validated by Kroenke, Spitzer, Williams, & Löwe (2009) [44], is a 4-item questionnaire. It was well-designed for the purpose of accurately measuring elements of anxiety and depression. It considers the two-item measure for depression (PHQ–2), consisting of core criteria for depression, which has shown excellent operating characteristics for assessing depressive and anxiety disorders [45, 46]. It also includes a two-item measure for anxiety (GAD–2), which has also been shown to be a good screening tool for panic, social anxiety, and posttraumatic stress disorders [45]. this scale was validated by a number of peer-reviewed research articles [47, 48]. We’ve used the Arabic translation of this scale made and evaluated by Sahar Obeid et al. [49], .

Each of the four questions is answered on a four-point Likert type scale (“1 = Not at all” “2 = Several days”, “3 = More than half the days”, “4 = Nearly every day”). Total score is determined by adding together the scores of each of the 4 items. scores are rated as normal (0–2), mild [3–5], moderate [6–8], and severe [9–12] A score greater than 3 in the first two suggests anxiety, whereas in the last two items it suggests depression.

### Statistical analysis

Data was collected using Microsoft excel 2016. Analysis and cross-tabulation were performed using IBM SPSS V27.0. Descriptive statistics were performed for variables and a cross-tabulation was performed to study the association between stress caused by different subscales of MSSQ and MSPSS and demographic data. A spearman correlation test was used to determine the relation between the scales of MSSQ, MSPSS and PHQ-4 due to its suitability for assessing monotonic relationships between ranked variables. This non-parametric method allows for the evaluation of the strength and direction of the association between the three scales used in this research. Cronbach’s Alpha coefficient was calculated for the scales to measure their internal consistency. Chi-square independence test was conducted to assess the association between variables as Chi-square test is used to examine the relationships between categorical variables and to provide insight into the dependence and independence of the variables in question.Fisher’s exact test was conducted whenever the expected cell count is less than five; the test was two-tailed and values less than 0.05 were considered statistically significant.

## Results

### Descriptive sociodemographic statistics

The sociodemographic characteristics of the sample are reported in Table1.

**Table 1:** Sociodemographic data

Gender	
Male	417(34.3%)
Female	800(65.7%)
**Medical faculty**	
Medicine	511(42.0%)
Dentistry	338(27.8%)
Pharmacy	368(30.2%)
**Academic year**	
Second	418(34.3%)
Third	258(21.2%)
fourth	285(23.4%)
Fifth	215(17.7%)
Sixth	41(3.4%)
**Financial status**	
Low	54(4.4%)
Medium	407(33.4%)
Good	677(55.6%)
Ideal	79(6.5%)
**Source of** **income**	
Parents	1061(87.2%)
Freelance	100(8.2%)
Other	56(4.6%)
**Grade of last** **Semester**	
≤ 70	138(11.3%)
70-79.99	448(36.8%)
80-89.99	539(44.3%)
≥ 90	92(7.6%)

Out of the total responses, 65.7% were females. The academic year with the greatest number of responses was the second year with a percentage of 34.3%. On the other hand, the academic year with the least number of responses was the sixth year with a percentage of 3.4%. Most of the respondents were medical students (n = 511,42.0%), followed by pharmacy students (n = 368,30.2%) and dental students (n = 338,27.8%). Of participants, 65.2% lived inside Damascus and 61.5% lived in a rural environment while the rest were in an urban environment. When asked about their financial status, 55.6% of the students answered with “good” and 33.4% with” medium” while only 4,4% answered with “low” and 6.6% with “Ideal”. Almost all of the participants were not married (98.2%), and were non-smokers (87.7%).

When the students were asked about their parents’ level of education, their answers varied. These answers are displayed in Table 2.

**Table 2:** Parents level of education

Level of education	Mother	Father
Elementary	137(11.3%)	136(11.2%)
High school	284 (23.3%)	252(20.7%)
Institute degree	581 (47.7%)	676(55.5%)
University degree	215 (17.7%)	153(12.6%)

### Percieved stress

We have found out that the majority of the participants had moderate levels of stress (*n* = 630,51.8%). There was a strong correlation between gender and experiencing stresses related to academic matters, social events, and group events and interpersonal and teaching and learning interactions; females were found to have the mentioned domains as main sources of stress in higher rates than males (see supplementary Tables 1–7, Additional File 1).

Contrary to expectations, having another doctor as a family member had no correlation with the experienced stress in any subscale. No significant relation between being a smoker and having higher levels of stress was found except for ARS and GARS.

Interesting relations between the mother’s level of education and the MSSQ result, TLRS and GARS were found. However, no such relations were found considering the father’s level of education. We also found that marital status had no correlation with experienced levels of stress. The only relation we found between the year of study and stress was regarding drive and desire related stresses; most students from the third, fourth and the sixth year had moderate levels of stress regarding DRS while most students in the second and the fifth year had mild levels of stress.

We also found a relation between the field of study; medicine, dentistry or pharmacy, and academic-related stressors. A stronger relation was found between the field on one hand, and interpersonal and intrapersonal related stressors and group activity related stressors, on the other hand.

Nonetheless, grades of last semester were only significantly related to the teaching and learning related stressors.

The economic status was correlated with stress from drive and desire stressors as well as group activities stressors; none of the students who identified their household income as “ideal” had severe stress in DRS while students from low-income households were found to be suffering the most of high levels of stress resulted by DRS.

This pattern was repeated in the GARS scale. The value of Cronbach’s Alpha for MSSQ was α = 0.937.

### Perceived social support

There was a relation between the total score of MSPSS and gender where female students received more support than their male peers. We found a correlation between gender on one hand, and received social support from family and friends, and the existence of a trustworthy person the student can rely on, on the other hand.We also noticed a relation between the level of income and social support levels that come from family. However, there was no relation between the grades of last semester with MSPSS scale (P-value = 0.189) or any of its subscales.

When asked about the close person they can emotionally rely on, the most recurring answer that students mentioned was a friend (*n* = 455, 38.30%). The second most common answer was the student’s mother (*n* = 367, 30.89%), whereas mentioning any member of the extended family was the least recurring answer (*n* = 24, 2.02%).

There was no significant relation between the medical faculty and the scores of MSPSS or any of its subscales (see supplementary Tables 8–11, Additional File 1).

Also, an unexpected relation was found between the father’s level of education and the score of MSPSS. Another significant result was the existence of a relation between the faculty and the MSPSS subscale of having a close person. The value of Cronbach’s Alpha for MSPSS was α = 0.932.

### Depression and anxiety

Female students scored significantly higher than male students in all aspects of the PHQ-4 scale (see supplementary Tables 12–14, Additional File 1), which suggests them having more depressive and anxiety symptoms than their male peers The only subscale which showed any correlation to the faculty of the student was anxiety. It is also worth mentioning that it was the only one that had a relation with the score of the last semester.

Nonetheless, the marital status was found to have a relation with the level of depression.

measured by PHQ-4. Furthermore, we’ve found that there was a difference between.

married and single students were more than half of the single students have symptomsof depression.

Moreover, students from inside Damascus were found to suffer from anxiety more than students from outside Damascus. The value of Cronbach’s Alpha for PHQ-4 was α = 0.850.

### Correlations between scales

As the data shown in table-3 suggests, when spearman’s correlation test was conducted there was a significant negative relationship between perceived social support and PHQ-4 score, *r*_s_(1215) = -0.163, *P* < .001. there was also a significant positive relationship between depressive symptoms and stress, *r*_s_(1215) = 0.349, *P* < .001. No correlation was found between MSPSS and MSSQ scale.

**Table 3:** Shows the correlations between stressors, social support, stress and anxiety

			MSSQ	MSPSS	PHQ4
Spearman’s rho	MSSQ	Correlation Coefficient	1.000	0.042	0.349^**^
		Sig. (2-tailed)	.	0.140	< 0.001
	MSPSS	Correlation Coefficient	0.042	1.000	− 0.163^**^
		Sig. (2-tailed)	0.140	.	< 0.001
	PHQ4	Correlation Coefficient	0.349^**^	− 0.163^**^	1.000
		Sig. (2-tailed)	< 0.001	< 0.001	.

**. Correlation is significant at the 0.01 level (2-tailed)

## Discussion

Medical schools, along with dentistry and pharmacy, demand heavy effort and impose stress onto the student’s everyday life. Therefore, getting social support from positive relations and family members is vital to reducing the amount of stress and improving the mental health of students [50] .

The results we acquired showed that the vast majority of students had moderate to high levels of stress (86.03%). Academic-related stressors were the most prevalent. Another study which was also conducted in Syria by Al Houri et al. [33], came to the same findings considering the demanding medical curriculum and excessive study times as contribution factors for this result along with the competition between students. Many other studies in different countries and regions reached the same result [51–57].

Despite having more social support from family and peers, we’ve found that ARS, TLRS, GARS and IRS were more prevalent in female students than male students. In addition to that, female students had more depressive and anxiety symptoms. We believe that one possible reason for this is that females are more socially allowed to express themselves and are more open to asking for and getting aid and support from their friends, which may allow them to have more social support from their family and friends than their male peers.This is confirmed by previous literature [12, 13]. However, Thompson G et al., as well as Al Houri et al., noticed no difference in social support between males and females [33, 58].

Nonetheless, Mary Thomas et al. [59] reported that females experience more clinical levels of imposter syndrome compared to males, which could be associated with experiencing more stress in different domains. Furthermore, females have also shown higher levels of self-oriented critical perfectionism [60], and as career stress may mediate the relation between perfectionism and depression [61], more females were found to report symptoms of depression and anxiety than males. This corresponds with previous literature [33, 52, 54, 62–64]. In contrast, a study in Bangladesh showed no difference between males and females regarding stress [51]. We hypothesize that the different results between males and females in our study may be related to the cultural traits of our society, which may not allow males to show feelings or ask for help easily.

We haven’t found any relation between the year of study and sources of stress; we expect that the reason behind this is that although clinical years can impose challenging obstacles as some studies suggest [53, 54, 62], the heavy curriculum in Damascus University along with the short amount of time to study it in the pre-clinical years can have as much of an association with stress as the hardships that come with clinical years.

Smokers were found to suffer less academic stress and stress from group-activities related stress. That can be justified by taking into account that students who are smokers are more likely to engage in non-academic activities that include their group of friends which may result in drifting their focus away from their studies. Positive correlation between smoking and stress was recorded in a previous article in the US [65].

Medical students were found to suffer from severe stress regarding academic stressors and group activities in comparison with dentistry and pharmacy students. That probably is a result of the heavy curriculum that medical school has, compared to pharmacy and dentistry. While dentistry students are the most likely to report severe stress reportedly caused by IRS, TLRS, and SRS, we believe that this may be a natural consequence of the highly collaborative environment that requires a high degree of collaboration between students. This, in turn, demands good communication skills and may be associated with greater levels of stress regarding learning and relations with peers compared to the single-student assignments that are more common in medical and pharmacy faculties. That might provide an explanation on why we recorded higher results in depression and anxiety among dental students compared to medicine and pharmacy. However, dental students’ future career is more defined than medical students -who have much longer years in specialization- and pharmacy students -who have to choose between a variety of working environments- which may explain how dentistry students were the least to suffer from severe levels of stress caused by drive and desire stressors.

Finding that the grades of the last semester were not associated with most scales and subscales was not expected because it indicated that social support had no apparent association, regardless of whether it was from family or friends, which **is consistent with** the null hypothesis in our article. This outcome differed from what some previous studies reported [28–30, 66]. However, Rospenda, k M et al. [67], came to the same results in a study conducted on 153 medical students in the USA. This led us to consider the possibility that the students in our sample did not need support from their environment to enhance their academic performance and that they depended mainly on intrinsic factors within themselves rather than external support, or regardless of whether such support existed or not. This might be a psychological observation potentially linked **to** the grinding civil war in Syria that affected these students’ childhood or other factors that were beyond the scope of this study. Consequently, more research is advised on this topic to uncover the main factors affecting stress in medical students and their performance. This was further supported by the fact that perceived stress was independent from social support in our sample. We also found a negative correlation between grades and anxiety which is an expected relation. The more anxiety students reported, the less they tended to report concentrating on their studies “A friend” was the most prevalent answer for the person to whom the students go seeking support. This might suggest that the students’ social orientation is more extroverted and informal with colleagues. However, further research is needed to study the social relations that medical students have and whether their studies significantly correlate with these relations.

This implies the importance of friendships on one hand and the need to encourage more students to reach out for help through implementing peer support programs and workshops to raise awareness and to destigmatize discussing mental health openly. Other implications include a review of the curriculum to maintain the academic rigor but also leave room for personal development and even academically teaching students proper ways to take care of their mental health and that of their close friends as a way of first-responding to psychologic crises.

## Conclusion

The primary aim of our study was to explore the relationship between the social support received by our sample and their academic performance and mental well-being. One of our key findings revealed that the grades of our sample for the last semester were not significantly influenced by any variables related to social support. This suggests that support from family or friends did not have a notable impact on academic performance. However, we also found a negative correlation between perceived social support and reported depressive symptoms among students.

In light of these findings, we encourage educational institutions to implement social support programs that provide students with the necessary resources to maintain their mental well-being. Although social support was not found to be significantly associated with academic grades in this study, it may be linked to enhanced mental health. We recommend the establishment of networks within universities and institutions to facilitate peer support, particularly for students in the same year or younger. This platform could serve as a vital resource for psychological first aid, especially in a society where access to adequate healthcare has been severely limited due to the economic challenges faced over the past 14 years. We remain hopeful that these obstacles will be addressed in the near future.

Furthermore, we advocate for additional research into other forms of support and their potential association with students’ academic performance. By expanding our understanding of these dynamics, we can better equip students to thrive both academically and psychologically.

### Limitations

A main limitation we faced was the small number of smokers because almost all of the participants were non-smokers (87.7%), which might make the results related to smoking inaccurate and not as reliable as the rest of our results. So, it was difficult to examine the assumptions for statistical modeling. Wider research, including a bigger sample with enough smokers, would help in checking the validity of our findings regarding this group. The same is applicable for married people among our sample because marriage at this age is not common in the study community, they do not represent a big portion of our research population (only 1.8%); this was reflected in our sample. Therefore, the results cannot be accurately generalized throughout the entire married population. More research is advised to check the findings regarding the effect of the student’s marital status on their stresses, depressive symptoms, academic scores, and the support the student received, especially in Syria as similar research had already been conducted in many other countries. More research can also be done to figure out whether academic scores and social support also had no relation in populations of students in other countries and/or other faculties and universities. We’ve recorded a relatively small number of six-year students’ participations due to their upcoming exams; this might have interfered with our result regarding correlation between the year of study and different scales.

The data might have been subject to self-report biases such as recall bias regarding the last semester’s average, or bias in measurement methods such as estimating financial status, or other response biases that can occur due to misunderstandings or having a tendency to answer in a particular manner and some exposures may not have been consistent throughout the study period such as smoking or place of residence. Also, students in the sixth year of their studies represented a lower proportion in the sample in comparison to younger students due to the absence of obligatory classes in the final year of medical education, so the results of depression and different scales among them may have been more accurate if the proportions were equal.

## Supplementary Information

Below is the link to the electronic supplementary material.

Supplementary Material 1

## Data Availability

The datasets used are available from the corresponding author upon reasonable request.

## Abbreviations

MSPSS: Multidimensional Scale of Perceived Stress Scale
PHQ-4: The Four-Item Patient Health Questionnaire
MSSQ: Medical Student Stress Scale
ARS: Academic Related Stressor
IRS: Interpersonal & Intrapersonal Related Stressor
TLRS: Teaching and Learning Related Stressor
SRS: Social Related Stressor
DRS: Drive & Desire Related Stressor
GARS: Group Activities Related Stressor
