# Supplementary material for: The cherry on top: nice but necessary? The impact of social support on perceived stress, depressive symptoms, and academic performance among medical students at Damascus university
Source: BMC Psychol. 2025 Sep 30;13:1092. doi: 10.1186/s40359-025-03329-0 (PMC12487554; doi:10.1186/s40359-025-03329-0)
Supplement: Supplementary file 1 — Supplementary Material 1 [file 40359_2025_3329_MOESM1_ESM.docx]

**Appendix for a Research Article**

| Table-1 Cross-Tabulation of MSPSS with Demographics | | | | | | | |  |  |  |
| --- | --- | --- | --- | --- | --- | --- | --- | --- | --- | --- |
|  | | MSPSS | | | | | | Test Value | Df | P Value |
|  |  | Low Support | | Moderate Support | | High Support | |  |  |  |
| Sex | Male | 60 | 14% | 152 | 36% | 205 | 49% | 12.260a | 2 | 0.002 |
|  | Female | 82 | 10% | 243 | 30% | 475 | 59% |  |  |  |
| Does He / She Smoke? | Doesn’t Smoke | 128 | 12% | 345 | 32% | 594 | 56% | 1.312a | 2 | 0.519 |
|  | Smokes | 13 | 9% | 50 | 34% | 85 | 57% |  |  |  |
| Is Any of Your Parents a doctor? | Yes | 13 | 8% | 43 | 28% | 98 | 64% | 4.591a | 2 | 0.101 |
|  | No | 129 | 12% | 352 | 33% | 582 | 55% |  |  |  |
| Mother's Level of Education | Primary | 19 | 14% | 55 | 40% | 63 | 46% | 10.640a | 6 | 0.1 |
|  | Secondary | 35 | 12% | 79 | 28% | 170 | 60% |  |  |  |
|  | College | 59 | 10% | 196 | 34% | 326 | 56% |  |  |  |
|  | Associate Degree | 29 | 13% | 65 | 30% | 121 | 56% |  |  |  |
| Father’s Level of Education | Primary | 24 | 18% | 51 | 38% | 61 | 45% | 16.924a | 6 | 0.01 |
|  | Secondary | 30 | 12% | 77 | 31% | 145 | 58% |  |  |  |
|  | College | 72 | 11% | 204 | 30% | 400 | 59% |  |  |  |
|  | Associate Degree | 16 | 10% | 63 | 41% | 74 | 48% |  |  |  |
| Marital Status | Lonely | 141 | 12% | 387 | 32% | 667 | 56% | 1.121a | 2 | 0.571 |
|  | Married | 1 | 5% | 8 | 36% | 13 | 59% |  |  |  |
| Study Year | Second | 44 | 11% | 131 | 31% | 243 | 58% | 10.620a | 8 | 0.224 |
|  | Third | 38 | 15% | 90 | 35% | 130 | 50% |  |  |  |
|  | Fourth | 26 | 9% | 98 | 34% | 161 | 56% |  |  |  |
|  | Fifth | 27 | 13% | 68 | 32% | 120 | 56% |  |  |  |
|  | Sixth | 7 | 17% | 8 | 20% | 26 | 63% |  |  |  |
| Medical College | Medicine | 68 | 13% | 154 | 30% | 289 | 57% | 6.806a | 4 | 0.147 |
|  | Dentistry | 40 | 12% | 105 | 31% | 193 | 57% |  |  |  |
|  | Pharmacy | 34 | 9% | 136 | 37% | 198 | 54% |  |  |  |
| Grades of Last Semester | <70 | 23 | 17% | 44 | 32% | 71 | 51% | 8.738a | 6 | 0.189 |
|  | 70-79.99 | 50 | 11% | 154 | 34% | 244 | 54% |  |  |  |
|  | 80-89.99 | 57 | 11% | 162 | 30% | 320 | 59% |  |  |  |
|  | >=90 | 12 | 13% | 35 | 38% | 45 | 49% |  |  |  |
| Had Any Volunteer Experience | Yes | 57 | 10% | 165 | 30% | 322 | 59% | 4.500a | 2 | 0.105 |
|  | No | 85 | 13% | 230 | 34% | 358 | 53% |  |  |  |
| Place of Residence | City | 79 | 11% | 246 | 33% | 423 | 57% | 2.306a | 2 | 0.316 |
|  | Countryside | 63 | 13% | 149 | 32% | 257 | 55% |  |  |  |
|  | Inside Damascus | 83 | 10% | 278 | 35% | 432 | 54% | 8.353a | 2 | 0.015 |
|  | Outside Damascus | 59 | 14% | 117 | 28% | 248 | 58% |  |  |  |
| Sharing Residence with: | Parents | 117 | 11% | 342 | 33% | 564 | 55% | 4.713a | 6 | 0.581 |
|  | College | 11 | 12% | 27 | 29% | 54 | 59% |  |  |  |
|  | Rented | 9 | 13% | 16 | 23% | 46 | 65% |  |  |  |
|  | Relative | 5 | 16% | 10 | 32% | 16 | 52% |  |  |  |
| Economic Status | Low | 11 | 20% | 18 | 33% | 25 | 46% | 13.414a | 6 | 0.037 |
|  | Middle | 58 | 14% | 140 | 34% | 209 | 51% |  |  |  |
|  | Good | 65 | 10% | 215 | 32% | 397 | 59% |  |  |  |
|  | Perfect | 8 | 10% | 22 | 28% | 49 | 62% |  |  |  |
| Income | Parent | 124 | 12% | 338 | 32% | 599 | 56% | 6.209a | 8 | 0.624 |
|  | Private | 15 | 15% | 33 | 33% | 52 | 52% |  |  |  |
|  | Other | 2 | 9% | 9 | 39% | 12 | 52% |  |  |  |
|  | Both | 1 | 3% | 14 | 47% | 15 | 50% |  |  |  |

| Table-2 Cross-Tabulation of Significant Other Subscale of MSPSS with Demographics | | | | | | | | | | |
| --- | --- | --- | --- | --- | --- | --- | --- | --- | --- | --- |
| MSPSS-Significant Other | | | | | | | | | | |
|  | | Low Support | | Moderate Support | | High Support | | Test Value | Df | P Value |
| Sex | Male | 72 | 17% | 127 | 30% | 218 | 52% | 15.943a | 2 | <0.001 |
|  | Female | 80 | 10% | 228 | 29% | 492 | 62% |  |  |  |
| Does He / She Smoke? | Doesn’t Smoke | 139 | 13% | 309 | 29% | 619 | 58% | 2.905a | 2 | 0.234 |
|  | Smokes | 12 | 8% | 46 | 31% | 90 | 61% |  |  |  |
| Is Any of Your Parents a doctor? | Yes | 13 | 8% | 43 | 28% | 98 | 64% | 3.255a | 2 | 0.196 |
|  | No | 139 | 13% | 312 | 29% | 612 | 58% |  |  |  |
| Mother's Level of Education | Primary | 17 | 12% | 54 | 39% | 66 | 48% | 9.491a | 6 | 0.148 |
|  | Secondary | 35 | 12% | 82 | 29% | 167 | 59% |  |  |  |
|  | College | 76 | 13% | 155 | 27% | 350 | 60% |  |  |  |
|  | Associate Degree | 24 | 11% | 64 | 30% | 127 | 59% |  |  |  |
| Father’s Level of Education | Primary | 27 | 20% | 41 | 30% | 68 | 50% | 15.035a | 6 | 0.02 |
|  | Secondary | 27 | 11% | 81 | 32% | 144 | 57% |  |  |  |
|  | College | 80 | 12% | 179 | 26% | 417 | 62% |  |  |  |
|  | Associate Degree | 18 | 12% | 54 | 35% | 81 | 53% |  |  |  |
| Marital Status | Lonely | 152 | 13% | 347 | 29% | 696 | 58% | 3.304a | 2 | 0.192 |
|  | Married | 0 | 0% | 8 | 36% | 14 | 64% |  |  |  |
| Study Year | Second | 53 | 13% | 117 | 28% | 248 | 59% | 10.398a | 8 | 0.238 |
|  | Third | 39 | 15% | 79 | 31% | 140 | 54% |  |  |  |
|  | Fourth | 24 | 8% | 91 | 32% | 170 | 60% |  |  |  |
|  | Fifth | 28 | 13% | 60 | 28% | 127 | 59% |  |  |  |
|  | Sixth | 8 | 20% | 8 | 20% | 25 | 61% |  |  |  |
| Medical College | Medicine | 88 | 17% | 124 | 24% | 299 | 59% | 26.779a | 4 | <0.001 |
|  | Dentistry | 39 | 12% | 106 | 31% | 193 | 57% |  |  |  |
|  | Pharmacy | 25 | 7% | 125 | 34% | 218 | 59% |  |  |  |
| Grades of Last Semester | <70 | 20 | 14% | 39 | 28% | 79 | 57% | 9.947a | 6 | 0.127 |
|  | 70-79.99 | 48 | 11% | 145 | 32% | 255 | 57% |  |  |  |
|  | 80-89.99 | 68 | 13% | 140 | 26% | 331 | 61% |  |  |  |
|  | >=90 | 16 | 17% | 31 | 34% | 45 | 49% |  |  |  |
| Had Any Volunteer Experience | Yes | 68 | 13% | 137 | 25% | 339 | 62% | 8.025a | 2 | 0.018 |
|  | No | 84 | 12% | 218 | 32% | 371 | 55% |  |  |  |
| Place of Residence | City | 95 | 13% | 219 | 29% | 434 | 58% | .111a | 2 | 0.946 |
|  | Countryside | 57 | 12% | 136 | 29% | 276 | 59% |  |  |  |
|  | Inside Damascus | 95 | 12% | 240 | 30% | 458 | 58% | 1.542a | 2 | 0.462 |
|  | Outside Damascus | 57 | 13% | 115 | 27% | 252 | 59% |  |  |  |
| Sharing Residence with: | Parents | 134 | 13% | 289 | 28% | 600 | 59% | 6.956a | 6 | 0.325 |
|  | College | 9 | 10% | 34 | 37% | 49 | 53% |  |  |  |
|  | Rented | 4 | 6% | 23 | 32% | 44 | 62% |  |  |  |
|  | Relative | 5 | 16% | 9 | 29% | 17 | 55% |  |  |  |
| Economic Status | Low | 12 | 22% | 14 | 26% | 28 | 52% | 9.249a | 6 | 0.16 |
|  | Middle | 58 | 14% | 123 | 30% | 226 | 56% |  |  |  |
|  | Good | 74 | 11% | 198 | 29% | 405 | 60% |  |  |  |
|  | Perfect | 8 | 10% | 20 | 25% | 51 | 65% |  |  |  |
| Income | Parent | 137 | 13% | 311 | 29% | 613 | 58% | 7.619a | 8 | 0.472 |
|  | Private | 14 | 14% | 29 | 29% | 57 | 57% |  |  |  |
|  | Other | 1 | 4% | 6 | 26% | 16 | 70% |  |  |  |
|  | Both | 0 | 0% | 8 | 27% | 22 | 73% |  |  |  |

| Table-3 Cross-Tabulation of Family Subscale of MSPSS with Demographics | | | | | | | | | | |
| --- | --- | --- | --- | --- | --- | --- | --- | --- | --- | --- |
|  | | MSPSS-Family | | | | | | Test Value | Df | P Value |
|  |  | Low Support | | Moderate Support | | High Support | |  |  |  |
| Sex | Male | 47 | 11% | 152 | 36% | 218 | 52% | 7.616a | 2 | 0.022 |
|  | Female | 81 | 10% | 236 | 30% | 483 | 60% |  |  |  |
| Does He / She Smoke? | Doesn’t Smoke | 114 | 11% | 337 | 32% | 616 | 58% | .649a | 2 | 0.723 |
|  | Smokes | 13 | 9% | 50 | 34% | 85 | 57% |  |  |  |
| Is Any of Your Parents a doctor? | Yes | 11 | 7% | 41 | 27% | 102 | 66% | 5.720a | 2 | 0.057 |
|  | No | 117 | 11% | 347 | 33% | 599 | 56% |  |  |  |
| Mother's Level of Education | Primary | 20 | 15% | 51 | 37% | 66 | 48% | 7.587a | 6 | 0.27 |
|  | Secondary | 29 | 10% | 84 | 30% | 171 | 60% |  |  |  |
|  | College | 54 | 9% | 186 | 32% | 341 | 59% |  |  |  |
|  | Associate Degree | 25 | 12% | 67 | 31% | 123 | 57% |  |  |  |
| Father's Level of Education | Primary | 21 | 15% | 47 | 35% | 68 | 50% | 7.744a | 6 | 0.257 |
|  | Secondary | 27 | 11% | 78 | 31% | 147 | 58% |  |  |  |
|  | College | 67 | 10% | 207 | 31% | 402 | 59% |  |  |  |
|  | Associate Degree | 13 | 8% | 56 | 37% | 84 | 55% |  |  |  |
| Marital Status | Lonely | 128 | 11% | 377 | 32% | 690 | 58% | 4.888a | 2 | 0.087 |
|  | Married | 0 | 0% | 11 | 50% | 11 | 50% |  |  |  |
| Study Year | Second | 41 | 10% | 133 | 32% | 244 | 58% | 6.582a | 8 | 0.582 |
|  | Third | 30 | 12% | 88 | 34% | 140 | 54% |  |  |  |
|  | Fourth | 25 | 9% | 89 | 31% | 171 | 60% |  |  |  |
|  | Fifth | 24 | 11% | 68 | 32% | 123 | 57% |  |  |  |
|  | Sixth | 8 | 20% | 10 | 24% | 23 | 56% |  |  |  |
| Medical College | Medicine | 59 | 12% | 162 | 32% | 290 | 57% | 4.088a | 4 | 0.394 |
|  | Dentistry | 37 | 11% | 98 | 29% | 203 | 60% |  |  |  |
|  | Pharmacy | 32 | 9% | 128 | 35% | 208 | 57% |  |  |  |
| Grades of Last Semester | <70 | 22 | 16% | 44 | 32% | 72 | 52% | 8.079a | 6 | 0.232 |
|  | 70-79.99 | 45 | 10% | 150 | 33% | 253 | 56% |  |  |  |
|  | 80-89.99 | 50 | 9% | 162 | 30% | 327 | 61% |  |  |  |
|  | >=90 | 11 | 12% | 32 | 35% | 49 | 53% |  |  |  |
| Had Any Volunteer Experience | Yes | 67 | 12% | 155 | 28% | 322 | 59% | 7.001a | 2 | 0.03 |
|  | No | 61 | 9% | 233 | 35% | 379 | 56% |  |  |  |
| Place of Residence | City | 76 | 10% | 239 | 32% | 433 | 58% | .266a | 2 | 0.875 |
|  | Countryside | 52 | 11% | 149 | 32% | 268 | 57% |  |  |  |
|  | Inside Damascus | 80 | 10% | 270 | 34% | 443 | 56% | 4.941a | 2 | 0.085 |
|  | Outside Damascus | 48 | 11% | 118 | 28% | 258 | 61% |  |  |  |
| Sharing Residence with: | Parents | 106 | 10% | 332 | 32% | 585 | 57% | 7.679a | 6 | 0.263 |
|  | College | 12 | 13% | 31 | 34% | 49 | 53% |  |  |  |
|  | Rented | 6 | 8% | 14 | 20% | 51 | 72% |  |  |  |
|  | Relative | 4 | 13% | 11 | 35% | 16 | 52% |  |  |  |
| Economic Status | Low | 11 | 20% | 20 | 37% | 23 | 43% | 18.232a | 6 | 0.006 |
|  | Middle | 51 | 13% | 143 | 35% | 213 | 52% |  |  |  |
|  | Good | 59 | 9% | 199 | 29% | 419 | 62% |  |  |  |
|  | Perfect | 7 | 9% | 26 | 33% | 46 | 58% |  |  |  |
| Income | Parent | 110 | 10% | 327 | 31% | 624 | 59% | 9.663a | 8 | 0.289 |
|  | Private | 9 | 9% | 41 | 41% | 50 | 50% |  |  |  |
|  | Other | 3 | 13% | 10 | 43% | 10 | 43% |  |  |  |
|  | Both | 6 | 20% | 9 | 30% | 15 | 50% |  |  |  |

| Table-4 Cross-Tabulation of Friends Subscale of MSPSS with Demographics | | | | | | | | | | |
| --- | --- | --- | --- | --- | --- | --- | --- | --- | --- | --- |
|  | | MSPSS-Friends | | | | | | Test Value | Df | P Value |
|  |  | Low Support | | Moderate Support | | High Support | |  |  |  |
| Sex | Male | 71 | 17% | 162 | 39% | 184 | 44% | 9.644a | 2 | 0.008 |
|  | Female | 129 | 16% | 247 | 31% | 424 | 53% |  |  |  |
| Does He / She Smoke? | Doesn’t Smoke | 180 | 17% | 361 | 34% | 526 | 49% | 2.134a | 2 | 0.344 |
|  | Smokes | 19 | 13% | 48 | 32% | 81 | 55% |  |  |  |
| Is Any of Your Parents a doctor? | Yes | 23 | 15% | 53 | 34% | 78 | 51% | .292a | 2 | 0.864 |
|  | No | 177 | 17% | 356 | 33% | 530 | 50% |  |  |  |
| Mother's Level of Education | Primary | 27 | 20% | 52 | 38% | 58 | 42% | 7.524a | 6 | 0.275 |
|  | Secondary | 39 | 14% | 100 | 35% | 145 | 51% |  |  |  |
|  | College | 92 | 16% | 194 | 33% | 295 | 51% |  |  |  |
|  | Associate Degree | 42 | 20% | 63 | 29% | 110 | 51% |  |  |  |
| Father’s Level of Education | Primary | 32 | (24% | 49 | 36% | 55 | 40% | 12.051a | 6 | 0.061 |
|  | Secondary | 41 | 16% | 82 | 33% | 129 | 51% |  |  |  |
|  | College | 101 | 15% | 218 | 32% | 357 | 53% |  |  |  |
|  | Associate Degree | 26 | 17% | 60 | 39% | 67 | 44% |  |  |  |
| Marital Status | Lonely | 199 | 17% | 398 | 33% | 598 | 50% | 3.809a | 2 | 0.149 |
|  | Married | 1 | 5% | 11 | 50% | 10 | 45% |  |  |  |
| Study Year | Second | 71 | 17% | 141 | 34% | 206 | 49% | 5.617a | 8 | 0.69 |
|  | Third | 46 | 18% | 93 | 36% | 119 | 46% |  |  |  |
|  | Fourth | 39 | 14% | 98 | 34% | 148 | 52% |  |  |  |
|  | Fifth | 38 | 18% | 62 | 29% | 115 | 53% |  |  |  |
|  | Sixth | 6 | 15% | 15 | 37% | 20 | 49% |  |  |  |
| Medical College | Medicine | 91 | 18% | 171 | 33% | 249 | 49% | 5.827a | 4 | 0.212 |
|  | Dentistry | 48 | 14% | 104 | 31% | 186 | 55% |  |  |  |
|  | Pharmacy | 61 | 17% | 134 | 36% | 173 | 47% |  |  |  |
| Grades of Last Semester | <70 | 28 | 20% | 46 | 33% | 64 | 46% | 6.697a | 6 | 0.35 |
|  | 70-79.99 | 67 | 15% | 151 | 34% | 230 | 51% |  |  |  |
|  | 80-89.99 | 85 | 16% | 177 | 33% | 277 | 51% |  |  |  |
|  | >=90 | 20 | 22% | 35 | 38% | 37 | 40% |  |  |  |
| Had Any Volunteer Experience | Yes | 178 | 33% | 292 | 54% | 74 | 14% | 7.749a | 2 | 0.021 |
|  | No | 231 | 34% | 316 | 47% | 126 | 19% |  |  |  |
| Place of Residence | City | 120 | 16% | 245 | 33% | 383 | 51% | 1.203a | 2 | 0.548 |
|  | Countryside | 80 | 17% | 164 | 35% | 225 | 48% |  |  |  |
|  | Inside Damascus | 123 | 16% | 281 | 35% | 389 | 49% | 3.816a | 2 | 0.148 |
|  | Outside Damascus | 77 | 18% | 128 | 30% | 219 | 52% |  |  |  |
| Sharing Residence with: | Parents | 168 | 16% | 346 | 34% | 509 | 50% | 1.410a | 6 | 0.965 |
|  | College | 15 | 16% | 29 | 32% | 48 | 52% |  |  |  |
|  | Rented | 10 | 14% | 24 | 34% | 37 | 52% |  |  |  |
|  | Relative | 7 | 23% | 10 | 32% | 14 | 45% |  |  |  |
| Economic Status | Low | 10 | 19% | 16 | 30% | 28 | 52% | 6.083a | 6 | 0.414 |
|  | Middle | 79 | 19% | 140 | 34% | 188 | 46% |  |  |  |
|  | Good | 101 | 15% | 226 | 33% | 350 | 52% |  |  |  |
|  | Perfect | 10 | 13% | 27 | 34% | 42 | 53% |  |  |  |
| Income | Parent | 178 | 17% | 350 | 33% | 533 | 50% | 4.296a | 8 | 0.83 |
|  | Private | 16 | 16% | 38 | 38% | 46 | 46% |  |  |  |
|  | Other | 3 | 13% | 7 | 30% | 13 | 57% |  |  |  |
|  | Both | 3 | 10% | 12 | 40% | 15 | 50% |  |  |  |

| Table-5 Cross-Tabulation of MSSQ scale with Demographics | | | | | | | | | | | | |
| --- | --- | --- | --- | --- | --- | --- | --- | --- | --- | --- | --- | --- |
|  | | MSSQ | | | | | | | | Test Value | Df | P-Value |
|  |  | Mild Stress | | Moderate Stress | | High Stress | | Severe Stress | |  |  |  |
| Sex | Male | 65 | 16% | 233 | 56% | 104 | 25% | 15 | 4% | 42.549a | 3 | <.001 |
|  | Female | 52 | 7% | 397 | 50% | 313 | 39% | 38 | 5% |  |  |  |
| Does He / She Smoke? | Doesn’t Smoke | 101 | 9% | 543 | 51% | 377 | 35% | 46 | 4% | 4.185a | 3 | 0.242 |
|  | Smokes | 16 | 11% | 86 | 58% | 40 | 27% | 6 | 4% |  |  |  |
| Is Any of Your Parents a doctor? | Yes | 14 | 9% | 82 | 53% | 52 | 34% | 6 | 4% | .223a | 3 | 0.974 |
|  | No | 103 | 10% | 548 | 52% | 365 | 34% | 47 | 4% |  |  |  |
| Mother's Level of Education | Primary | 13 | 9% | 67 | 49% | 51 | 37% | 6 | 4% | 22.971a | 9 | 0.006 |
|  | Secondary | 26 | 9% | 144 | 51% | 103 | 36% | 11 | 4% |  |  |  |
|  | College | 68 | 12% | 281 | 48% | 208 | 36% | 24 | 4% |  |  |  |
|  | Associate Degree | 10 | 5% | 138 | 64% | 55 | 26% | 12 | 6% |  |  |  |
| Father’s Level of Education | Primary | 11 | 8% | 75 | 55% | 45 | 33% | 5 | 4% | 13.072a | 9 | 0.159 |
|  | Secondary | 17 | 7% | 132 | 52% | 89 | 35% | 14 | 6% |  |  |  |
|  | College | 73 | 11% | 331 | 49% | 245 | 36% | 27 | 4% |  |  |  |
|  | Associate Degree | 16 | 10% | 92 | 60% | 38 | 25% | 7 | 5% |  |  |  |
| Marital Status | Lonely | 114 | 10% | 617 | 52% | 411 | 34% | 53 | 4% | 1.905a | 3 | 0.592 |
|  | Married | 3 | 14% | 13 | 59% | 6 | 27% | 0 | 0% |  |  |  |
| Study Year | Second | 51 | 12% | 217 | 52% | 132 | 32% | 18 | 4% | 12.668a | 12 | 0.394 |
|  | Third | 23 | 9% | 134 | 52% | 91 | 35% | 10 | 4% |  |  |  |
|  | Fourth | 25 | 9% | 136 | 48% | 107 | 38% | 17 | 6% |  |  |  |
|  | Fifth | 15 | 7% | 123 | 57% | 70 | 33% | 7 | 3% |  |  |  |
|  | Sixth | 3 | 7% | 20 | 49% | 17 | 41% | 1 | 2% |  |  |  |
| Medical College | Medicine | 57 | 11% | 259 | 51% | 170 | 33% | 25 | 5% | 11.769a | 6 | 0.067 |
|  | Dentistry | 24 | 7% | 169 | 50% | 125 | 37% | 20 | 6% |  |  |  |
|  | Pharmacy | 36 | 10% | 202 | 55% | 122 | 33% | 8 | 2% |  |  |  |
| Grades of Last Semester | <70 | 14 | 10% | 73 | 53% | 43 | 31% | 8 | 6% | 16.753a | 9 | 0.053 |
|  | 70-79.99 | 38 | 8% | 227 | 51% | 164 | 37% | 19 | 4% |  |  |  |
|  | 80-89.99 | 46 | 9% | 285 | 53% | 185 | 34% | 23 | 4% |  |  |  |
|  | >=90 | 19 | 21% | 45 | 49% | 25 | 27% | 3 | 3% |  |  |  |
| Had Any Volunteer Experience | Yes | 48 | 9% | 291 | 53% | 188 | 35% | 17 | 3% | 4.647a | 3 | 0.2 |
|  | No | 69 | 10% | 339 | 50% | 229 | 34% | 36 | 5% |  |  |  |
| Place of Residence | City | 78 | 10% | 392 | 52% | 249 | 33% | 29 | 4% | 3.049a | 3 | 0.384 |
|  | Countryside | 39 | 8% | 238 | 51% | 168 | 36% | 24 | 5% |  |  |  |
|  | Inside Damascus | 74 | 9% | 412 | 52% | 274 | 35% | 33 | 4% | .455a | 3 | 0.929 |
|  | Outside Damascus | 43 | 10% | 218 | 51% | 143 | 34% | 20 | 5% |  |  |  |
| Sharing Residence with: | Parents | 94 | 9% | 528 | 52% | 355 | 35% | 46 | 4% | 8.833a | 9 | 0.453 |
|  | College | 14 | 15% | 48 | 52% | 26 | 28% | 4 | 4% |  |  |  |
|  | Rented | 5 | 7% | 41 | 58% | 22 | 31% | 3 | 4% |  |  |  |
|  | Relative | 4 | 13% | 13 | 42% | 14 | 45% | 0 | 0% |  |  |  |
| Economic Status | Low | 8 | 15% | 22 | 41% | 19 | 35% | 5 | 9% | 14.326a | 9 | 0.111 |
|  | Middle | 35 | 9% | 197 | 48% | 154 | 38% | 21 | 5% |  |  |  |
|  | Good | 68 | 10% | 371 | 55% | 213 | 31% | 25 | 4% |  |  |  |
|  | Perfect | 6 | 8% | 40 | 51% | 31 | 39% | 2 | 3% |  |  |  |
| Income | Parent | 99 | 9% | 543 | 51% | 372 | 35% | 47 | 4% | 7.108a | 12 | 0.85 |
|  | Private | 12 | 12% | 56 | 56% | 28 | 28% | 4 | 4% |  |  |  |
|  | Other | 4 | 17% | 13 | 57% | 5 | 22% | 1 | 4% |  |  |  |
|  | Both | 2 | 7% | 17 | 57% | 10 | 33% | 1 | 3% |  |  |  |

| Table-6 Cross-Tabulation of Academic Related Stressors Subscale of MSSQ with Demographics | | | | | | | | | | | | |
| --- | --- | --- | --- | --- | --- | --- | --- | --- | --- | --- | --- | --- |
|  |  | ARS | | | | | | | | Test Value | Df | P-Value |
|  |  | Mild Stress | | Moderate Stress | | High Stress | | Severe Stress | |  |  |  |
| Sex | Male | 35 | 8% | 150 | 36% | 188 | 45% | 44 | 11% | 65.270a | 3 | <.001 |
|  | Female | 12 | 2% | 189 | 24% | 459 | 57% | 140 | 18% |  |  |  |
| Does He / She Smoke? | Doesn’t Smoke | 37 | 3% | 287 | 27% | 576 | 54% | 167 | 16% | 9.910a | 3 | 0.019 |
|  | Smokes | 10 | 7% | 52 | 35% | 70 | 47% | 16 | 11% |  |  |  |
| Is Any of Your Parents a doctor? | Yes | 6 | 4% | 45 | 29% | 81 | 53% | 22 | 14% | .210a | 3 | 0.976 |
|  | No | 41 | 4% | 294 | 28% | 566 | 53% | 162 | 15% |  |  |  |
| Mother's Level of Education | Primary | 3 | 2% | 40 | 29% | 74 | 54% | 20 | 15% | 11.860a | 9 | 0.221 |
|  | Secondary | 7 | 2% | 70 | 25% | 158 | 56% | 49 | 17% |  |  |  |
|  | College | 31 | 5% | 166 | 29% | 294 | 51% | 90 | 15% |  |  |  |
|  | Associate Degree | 6 | 3% | 63 | 29% | 121 | 56% | 25 | 12% |  |  |  |
| Father’s Level of Education | Primary | 2 | 1% | 40 | 29% | 69 | 51% | 25 | 18% | 8.276a | 9 | 0.507 |
|  | Secondary | 8 | 3% | 61 | 24% | 140 | 56% | 43 | 17% |  |  |  |
|  | College | 29 | 4% | 191 | 28% | 359 | 53% | 97 | 14% |  |  |  |
|  | Associate Degree | 8 | 5% | 47 | 31% | 79 | 52% | 19 | 12% |  |  |  |
| Marital Status | Lonely | 46 | 4% | 331 | 28% | 636 | 53% | 182 | 15% | 1.190a | 3 | 0.755 |
|  | Married | 1 | 5% | 8 | 36% | 11 | 50% | 2 | 9% |  |  |  |
| Study Year | Second | 17 | 4% | 123 | 29% | 223 | 53% | 55 | 13% | 17.158a | 12 | 0.144 |
|  | Third | 11 | 4% | 56 | 22% | 151 | 59% | 40 | 16% |  |  |  |
|  | Fourth | 9 | 3% | 71 | 25% | 154 | 54% | 51 | 18% |  |  |  |
|  | Fifth | 8 | 4% | 77 | 36% | 97 | 45% | 33 | 15% |  |  |  |
|  | Sixth | 2 | 5% | 12 | 29% | 22 | 54% | 5 | 12% |  |  |  |
| Medical College | Medicine | 22 | 4% | 132 | 26% | 261 | 51% | 96 | 19% | 15.618a | 6 | 0.016 |
|  | Dentistry | 13 | 4% | 100 | 30% | 173 | 51% | 52 | 15% |  |  |  |
|  | Pharmacy | 12 | 3% | 107 | 29% | 213 | 58% | 36 | 10% |  |  |  |
| Grades of Last Semester | <70 | 6 | 4% | 39 | 28% | 70 | 51% | 23 | 17% | 12.691a | 9 | 0.177 |
|  | 70-79.99 | 16 | 4% | 123 | 27% | 253 | 56% | 56 | 13% |  |  |  |
|  | 80-89.99 | 17 | 3% | 149 | 28% | 284 | 53% | 89 | 17% |  |  |  |
|  | >=90 | 8 | 9% | 28 | 30% | 40 | 43% | 16 | 17% |  |  |  |
| Had Any Volunteer Experience | Yes | 19 | 3% | 152 | 28% | 304 | 56% | 69 | 13% | 5.577a | 3 | 0.134 |
|  | No | 28 | 4% | 187 | 28% | 343 | 51% | 115 | 17% |  |  |  |
| Place of Residence | City | 34 | 5% | 225 | 30% | 379 | 51% | 110 | 15% | 8.289a | 3 | 0.04 |
|  | Countryside | 13 | 3% | 114 | 24% | 268 | 57% | 74 | 16% |  |  |  |
|  | Inside Damascus | 28 | 4% | 231 | 29% | 420 | 53% | 114 | 14% | 2.822a | 3 | 0.42 |
|  | Outside Damascus | 19 | 4% | 108 | 25% | 227 | 54% | 70 | 17% |  |  |  |
| Sharing Residence with: | Parents | 35 | 3% | 274 | 27% | 558 | 55% | 156 | 15% | 20.047a | 9 | 0.018 |
|  | College | 6 | 7% | 34 | 37% | 43 | 47% | 9 | 10% |  |  |  |
|  | Rented | 4 | 6% | 27 | 38% | 30 | 42% | 10 | 14% |  |  |  |
|  | Relative | 2 | 6% | 4 | 13% | 16 | 52% | 9 | 29% |  |  |  |
| Economic Status | Low | 2 | 4% | 14 | 26% | 24 | 44% | 14 | 26% | 8.135a | 9 | 0.521 |
|  | Middle | 13 | 3% | 119 | 29% | 210 | 52% | 65 | 16% |  |  |  |
|  | Good | 28 | 4% | 184 | 27% | 369 | 55% | 96 | 14% |  |  |  |
|  | Perfect | 4 | 5% | 22 | 28% | 44 | 56% | 9 | 11% |  |  |  |
| Income | Parent | 42 | 4% | 286 | 27% | 565 | 53% | 168 | 16% | 10.084a | 12 | 0.609 |
|  | Private | 4 | 4% | 35 | 35% | 53 | 53% | 8 | 8% |  |  |  |
|  | Other | 1 | 4% | 9 | 39% | 9 | 39% | 4 | 17% |  |  |  |
|  | Both | 0 | 0% | 8 | 27% | 18 | 60% | 4 | 13% |  |  |  |

| Table-7 Cross-Tabulation of Interpersonal and Intrapersonal Related Stressors Subscale of MSSQ with Demographics | | | | | | | | | | | | |
| --- | --- | --- | --- | --- | --- | --- | --- | --- | --- | --- | --- | --- |
|  |  | IRS | | | | | | | | Test Value | Df | P-Value |
|  |  | Mild Stress | | Moderate Stress | | High Stress | | Severe Stress | |  |  |  |
| Sex | Male | 151 | 36% | 129 | 31% | 99 | 24% | 38 | 9% | 8.984a | 3 | 0.03 |
|  | Female | 236 | 30% | 236 | 30% | 243 | 30% | 85 | 11% |  |  |  |
| Does He / She Smoke? | Doesn’t Smoke | 335 | 31% | 320 | 30% | 299 | 28% | 113 | 11% | 2.466a | 3 | 0.481 |
|  | Smokes | 52 | 35% | 44 | 30% | 42 | 28% | 10 | 7% |  |  |  |
| Is Any of Your Parents a doctor? | Yes | 47 | 31% | 58 | 38% | 36 | 23% | 13 | 8% | 5.434a | 3 | 0.143 |
|  | No | 340 | 32% | 307 | 29% | 306 | 29% | 110 | 10% |  |  |  |
| Mother's Level of Education | Primary | 42 | 31% | 44 | 32% | 37 | 27% | 14 | 10% | 3.324a | 9 | 0.95 |
|  | Secondary | 96 | 34% | 85 | 30% | 71 | 25% | 32 | 11% |  |  |  |
|  | College | 183 | 31% | 175 | 30% | 169 | 29% | 54 | 9% |  |  |  |
|  | Associate Degree | 66 | 31% | 61 | 28% | 65 | 30% | 23 | 11% |  |  |  |
| Father’s Level of Education | Primary | 49 | 36% | 31 | 23% | 41 | 30% | 15 | 11% | 10.821a | 9 | 0.288 |
|  | Secondary | 69 | 27% | 81 | 32% | 74 | 29% | 28 | 11% |  |  |  |
|  | College | 216 | 32% | 199 | 29% | 195 | 29% | 66 | 10% |  |  |  |
|  | Associate Degree | 53 | 35% | 54 | 35% | 32 | 21% | 14 | 9% |  |  |  |
| Marital Status | Lonely | 381 | 32% | 358 | 30% | 335 | 28% | 121 | 10% | .302a | 3 | 0.96 |
|  | Married | 6 | 27% | 7 | 32% | 7 | 32% | 2 | 9% |  |  |  |
| Study Year | Second | 150 | 36% | 109 | 26% | 118 | 28% | 41 | 10% | 14.161a | 12 | 0.291 |
|  | Third | 85 | 33% | 80 | 31% | 69 | 27% | 24 | 9% |  |  |  |
|  | Fourth | 83 | 29% | 82 | 29% | 87 | 31% | 33 | 12% |  |  |  |
|  | Fifth | 55 | 26% | 80 | 37% | 59 | 27% | 21 | 10% |  |  |  |
|  | Sixth | 14 | 34% | 14 | 34% | 9 | 22% | 4 | 10% |  |  |  |
| Medical College | Medicine | 192 | 38% | 148 | 29% | 122 | 24% | 49 | 10% | 29.024a | 6 | <.001 |
|  | Dentistry | 77 | 23% | 101 | 30% | 113 | 33% | 47 | 14% |  |  |  |
|  | Pharmacy | 118 | 32% | 116 | 32% | 107 | 29% | 27 | 7% |  |  |  |
| Grades of Last Semester | <70 | 48 | 35% | 46 | 33% | 26 | 19% | 18 | 13% | 13.282a | 9 | 0.15 |
|  | 70-79.99 | 137 | 31% | 129 | 29% | 137 | 31% | 45 | 10% |  |  |  |
|  | 80-89.99 | 164 | 30% | 164 | 30% | 160 | 30% | 51 | 9% |  |  |  |
|  | >=90 | 38 | 41% | 26 | 28% | 19 | 21% | 9 | 10% |  |  |  |
| Had Any Volunteer Experience | Yes | 165 | 30% | 173 | 32% | 154 | 28% | 52 | 10% | 2.049a | 3 | 0.562 |
|  | No | 222 | 33% | 192 | 29% | 188 | 28% | 71 | 11% |  |  |  |
| Place of Residence | City | 247 | 33% | 225 | 30% | 203 | 27% | 73 | 10% | 1.789a | 3 | 0.617 |
|  | Countryside | 140 | 30% | 140 | 30% | 139 | 30% | 50 | 11% |  |  |  |
|  | Inside Damascus | 256 | 32% | 234 | 30% | 218 | 27% | 85 | 11% | 1.491a | 3 | 0.684 |
|  | Outside Damascus | 131 | 31% | 131 | 31% | 124 | 29% | 38 | 9% |  |  |  |
| Sharing Residence with: | Parents | 330 | 32% | 300 | 29% | 289 | 28% | 104 | 10% | 9.327a | 9 | 0.408 |
|  | College | 29 | 32% | 34 | 37% | 23 | 25% | 6 | 7% |  |  |  |
|  | Rented | 19 | 27% | 25 | 35% | 17 | 24% | 10 | 14% |  |  |  |
|  | Relative | 9 | 29% | 6 | 19% | 13 | 42% | 3 | 10% |  |  |  |
| Economic Status | Low | 18 | 33% | 17 | 31% | 12 | 22% | 7 | 13% | 15.712a | 9 | 0.073 |
|  | Middle | 107 | 26% | 126 | 31% | 135 | 33% | 39 | 10% |  |  |  |
|  | Good | 240 | 35% | 195 | 29% | 171 | 25% | 71 | 10% |  |  |  |
|  | Perfect | 22 | 28% | 27 | 34% | 24 | 30% | 6 | 8% |  |  |  |
| Income | Parent | 332 | 31% | 312 | 29% | 309 | 29% | 108 | 10% | 12.912a | 12 | 0.376 |
|  | Private | 35 | 35% | 34 | 34% | 21 | 21% | 10 | 10% |  |  |  |
|  | Other | 10 | 43% | 7 | 30% | 5 | 22% | 1 | 4% |  |  |  |
|  | Both | 10 | 33% | 9 | 30% | 7 | 23% | 4 | 13% |  |  |  |

| Table-8 Cross-Tabulation of Teaching and Learning Related Stressors Subscale of MSSQ with Demographics | | | | | | | | | | | | | |
| --- | --- | --- | --- | --- | --- | --- | --- | --- | --- | --- | --- | --- | --- |
|  |  | | TLRS | | | | | | | | Test Value | Df | P-Value |
|  |  |  | Mild Stress | | Moderate Stress | | High Stress | | Severe Stress | |  |  |  |
| Sex | | Male | 99 | 24% | 181 | 43% | 106 | 25% | 31 | 7% | 17.417a | 3 | 0.001 |
|  |  | Female | 120 | 15% | 345 | 43% | 257 | 32% | 78 | 10% |  |  |  |
| Does He / She Smoke? | | Doesn’t Smoke | 184 | 17% | 468 | 44% | 322 | 30% | 93 | 9% | 4.889a | 3 | 0.18 |
|  |  | Smokes | 35 | 24% | 58 | 39% | 39 | 26% | 16 | 11% |  |  |  |
| Is Any of Your Parents a doctor? | | Yes | 31 | 20% | 66 | 43% | 44 | 29% | 13 | 8% | .597a | 3 | 0.897 |
|  |  | No | 188 | 18% | 460 | 43% | 319 | 30% | 96 | 9% |  |  |  |
| Mother's Level of Education | | Primary | 28 | 20% | 59 | 43% | 39 | 28% | 11 | 8% | 20.842a | 9 | 0.013 |
|  |  | Secondary | 41 | 14% | 114 | 40% | 90 | 32% | 39 | 14% |  |  |  |
|  |  | College | 116 | 20% | 245 | 42% | 181 | 31% | 39 | 7% |  |  |  |
|  |  | Associate Degree | 34 | 16% | 108 | 50% | 53 | 25% | 20 | 9% |  |  |  |
| Father’s Level of Education | | Primary | 23 | 17% | 66 | 49% | 37 | 27% | 10 | 7% | 13.930a | 9 | 0.125 |
|  |  | Secondary | 37 | 15% | 109 | 43% | 74 | 29% | 32 | 13% |  |  |  |
|  |  | College | 127 | 19% | 278 | 41% | 217 | 32% | 54 | 8% |  |  |  |
|  |  | Associate Degree | 32 | 21% | 73 | 48% | 35 | 23% | 13 | 8% |  |  |  |
| Marital Status | | Lonely | 212 | 18% | 516 | 43% | 358 | 30% | 109 | 9% | 4.790a | 3 | 0.188 |
|  |  | Married | 7 | 32% | 10 | 45% | 5 | 23% | 0 | 0% |  |  |  |
| Study Year | | Second | 93 | 22% | 170 | 41% | 122 | 29% | 33 | 8% | 14.541a | 12 | 0.267 |
|  |  | Third | 39 | 15% | 113 | 44% | 84 | 33% | 22 | 9% |  |  |  |
|  |  | Fourth | 50 | 18% | 118 | 41% | 84 | 29% | 33 | 12% |  |  |  |
|  |  | Fifth | 32 | 15% | 103 | 48% | 63 | 29% | 17 | 8% |  |  |  |
|  |  | Sixth | 5 | 12% | 22 | 54% | 10 | 24% | 4 | 10% |  |  |  |
| Medical College | | Medicine | 105 | 21% | 215 | 42% | 146 | 29% | 45 | 9% | 7.915a | 6 | 0.244 |
|  |  | Dentistry | 49 | 14% | 143 | 42% | 110 | 33% | 36 | 11% |  |  |  |
|  |  | Pharmacy | 65 | 18% | 168 | 46% | 107 | 29% | 28 | 8% |  |  |  |
| Grades of Last Semester | | <70 | 29 | 21% | 57 | 41% | 35 | 25% | 17 | 12% | 17.050a | 9 | 0.048 |
|  |  | 70-79.99 | 65 | 15% | 202 | 45% | 137 | 31% | 44 | 10% |  |  |  |
|  |  | 80-89.99 | 105 | 19% | 224 | 42% | 173 | 32% | 37 | 7% |  |  |  |
|  |  | >=90 | 20 | 22% | 43 | 47% | 18 | 20% | 11 | 12% |  |  |  |
| Had Any Volunteer Experience | | Yes | 95 | 17% | 241 | 44% | 163 | 30% | 45 | 8% | .941a | 3 | 0.816 |
|  |  | No | 124 | 18% | 285 | 42% | 200 | 30% | 64 | 10% |  |  |  |
| Place of Residence | | City | 142 | 19% | 327 | 44% | 221 | 30% | 58 | 8% | 4.350a | 3 | 0.226 |
|  |  | Countryside | 77 | 16% | 199 | 42% | 142 | 30% | 51 | 11% |  |  |  |
|  | | Inside Damascus | 147 | 19% | 330 | 42% | 245 | 31% | 71 | 9% | 2.602a | 3 | 0.457 |
|  |  | Outside Damascus | 72 | 17% | 196 | 46% | 118 | 28% | 38 | 9% |  |  |  |
| Sharing Residence with: | | Parents | 177 | 17% | 442 | 43% | 309 | 30% | 95 | 9% | 9.001a | 9 | 0.437 |
|  |  | College | 19 | 21% | 43 | 47% | 23 | 25% | 7 | 8% |  |  |  |
|  |  | Rented | 19 | 27% | 26 | 37% | 23 | 32% | 3 | 4% |  |  |  |
|  |  | Relative | 4 | 13% | 15 | 48% | 8 | 26% | 4 | 13% |  |  |  |
| Economic Status | | Low | 11 | 20% | 22 | 41% | 14 | 26% | 7 | 13% | 8.353a | 9 | 0.499 |
|  |  | Middle | 64 | 16% | 170 | 42% | 131 | 32% | 42 | 10% |  |  |  |
|  |  | Good | 133 | 20% | 299 | 44% | 191 | 28% | 54 | 8% |  |  |  |
|  |  | Perfect | 11 | 14% | 35 | 44% | 27 | 34% | 6 | 8% |  |  |  |
| Income | | Parent | 190 | 18% | 449 | 42% | 328 | 31% | 94 | 9% | 10.542a | 12 | 0.569 |
|  |  | Private | 17 | 17% | 51 | 51% | 22 | 22% | 10 | 10% |  |  |  |
|  |  | Other | 6 | 26% | 11 | 48% | 4 | 17% | 2 | 9% |  |  |  |
|  |  | Both | 5 | 17% | 15 | 50% | 7 | 23% | 3 | 10% |  |  |  |

| Table-9 Cross-Tabulation of Social Related Stressors Subscale of MSSQ with Demographics | | | | | | | | | | | | | |
| --- | --- | --- | --- | --- | --- | --- | --- | --- | --- | --- | --- | --- | --- |
|  |  | | SRS | | | | | | | | Test Value | Df | P-Value |
|  |  |  | Mild Stress | | Moderate Stress | | High Stress | | Severe Stress | |  |  |  |
| Sex | | Male | 94 | 23% | 221 | 53% | 93 | 22% | 9 | 2% | 21.892a | 3 | <.001 |
|  |  | Female | 127 | 16% | 382 | 48% | 250 | 31% | 41 | 5% |  |  |  |
| Does He / She Smoke? | | Doesn’t Smoke | 197 | 18% | 517 | 48% | 307 | 29% | 46 | 4% | 4.505a | 3 | 0.212 |
|  |  | Smokes | 24 | 16% | 85 | 57% | 35 | 24% | 4 | 3% |  |  |  |
| Is Any of Your Parents a doctor? | | Yes | 36 | 23% | 70 | 45% | 43 | 28% | 5 | 3% | 3.562a | 3 | 0.313 |
|  |  | No | 185 | 17% | 533 | 50% | 300 | 28% | 45 | 4% |  |  |  |
| Mother's Level of Education | | Primary | 20 | 15% | 70 | 51% | 41 | 30% | 6 | 4% | 6.425a | 9 | 0.697 |
|  |  | Secondary | 53 | 19% | 138 | 49% | 83 | 29% | 10 | 4% |  |  |  |
|  |  | College | 113 | 19% | 277 | 48% | 168 | 29% | 23 | 4% |  |  |  |
|  |  | Associate Degree | 35 | 16% | 118 | 55% | 51 | 24% | 11 | 5% |  |  |  |
| Father’s Level of Education | | Primary | 20 | 15% | 73 | 54% | 38 | 28% | 5 | 4% | 13.989a | 9 | 0.123 |
|  |  | Secondary | 32 | 13% | 128 | 51% | 82 | 33% | 10 | 4% |  |  |  |
|  |  | College | 130 | 19% | 330 | 49% | 186 | 28% | 30 | 4% |  |  |  |
|  |  | Associate Degree | 39 | 25% | 72 | 47% | 37 | 24% | 5 | 3% |  |  |  |
| Marital Status | | Lonely | 218 | 18% | 591 | 49% | 336 | 28% | 50 | 4% | 1.391a | 3 | 0.708 |
|  |  | Married | 3 | 14% | 12 | 55% | 7 | 32% | 0 | 0% |  |  |  |
| Study Year | | Second | 94 | 22% | 205 | 49% | 107 | 26% | 12 | 3% | 15.343a | 12 | 0.223 |
|  |  | Third | 40 | 16% | 128 | 50% | 74 | 29% | 16 | 6% |  |  |  |
|  |  | Fourth | 51 | 18% | 140 | 49% | 81 | 28% | 13 | 5% |  |  |  |
|  |  | Fifth | 30 | 14% | 111 | 52% | 66 | 31% | 8 | 4% |  |  |  |
|  |  | Sixth | 6 | 15% | 19 | 46% | 15 | 37% | 1 | 2% |  |  |  |
| Medical College | | Medicine | 102 | 20% | 261 | 51% | 129 | 25% | 19 | 4% | 5.488a | 6 | 0.483 |
|  |  | Dentistry | 55 | 16% | 162 | 48% | 105 | 31% | 16 | 5% |  |  |  |
|  |  | Pharmacy | 64 | 17% | 180 | 49% | 109 | 30% | 15 | 4% |  |  |  |
| Grades of Last Semester | | <70 | 27 | 20% | 67 | 49% | 36 | 26% | 8 | 6% | 11.497a | 9 | 0.243 |
|  |  | 70-79.99 | 69 | 15% | 214 | 48% | 143 | 32% | 22 | 5% |  |  |  |
|  |  | 80-89.99 | 103 | 19% | 276 | 51% | 143 | 27% | 17 | 3% |  |  |  |
|  |  | >=90 | 22 | 24% | 46 | 50% | 21 | 23% | 3 | 3% |  |  |  |
| Had Any Volunteer Experience | | Yes | 93 | 17% | 281 | 52% | 150 | 28% | 20 | 4% | 2.071a | 3 | 0.558 |
|  |  | No | 128 | 19% | 322 | 48% | 193 | 29% | 30 | 4% |  |  |  |
| Place of Residence | | City | 149 | 20% | 367 | 49% | 202 | 27% | 30 | 4% | 4.406a | 3 | 0.221 |
|  |  | Countryside | 72 | 15% | 236 | 50% | 141 | 30% | 20 | 4% |  |  |  |
|  | | Inside Damascus | 150 | 19% | 394 | 50% | 216 | 27% | 33 | 4% | 1.463a | 3 | 0.691 |
|  |  | Outside Damascus | 71 | 17% | 209 | 49% | 127 | 30% | 17 | 4% |  |  |  |
| Sharing Residence with: | | Parents | 185 | 18% | 500 | 49% | 297 | 29% | 41 | 4% | 7.480a | 9 | 0.587 |
|  |  | College | 18 | 20% | 48 | 52% | 21 | 23% | 5 | 5% |  |  |  |
|  |  | Rented | 11 | 15% | 43 | 61% | 14 | 20% | 3 | 4% |  |  |  |
|  |  | Relative | 7 | 23% | 12 | 39% | 11 | 35% | 1 | 3% |  |  |  |
| Economic Status | | Low | 14 | 26% | 21 | 39% | 15 | 28% | 4 | 7% | 12.233a | 9 | 0.201 |
|  |  | Middle | 58 | 14% | 207 | 51% | 124 | 30% | 18 | 4% |  |  |  |
|  |  | Good | 134 | 20% | 336 | 50% | 180 | 27% | 27 | 4% |  |  |  |
|  |  | Perfect | 15 | 19% | 39 | 49% | 24 | 30% | 1 | 1% |  |  |  |
| Income | | Parent | 195 | 18% | 519 | 49% | 302 | 28% | 45 | 4% | 8.046a | 12 | 0.782 |
|  |  | Private | 15 | 15% | 55 | 55% | 26 | 26% | 4 | 4% |  |  |  |
|  |  | Other | 3 | 13% | 14 | 61% | 5 | 22% | 1 | 4% |  |  |  |
|  |  | Both | 7 | 23% | 15 | 50% | 8 | 27% | 0 | 0% |  |  |  |

| Table-10 Cross-Tabulation of Drive & Desire Related Stressors Subscale of MSSQ with Demographics | | | | | | | | | | | | |
| --- | --- | --- | --- | --- | --- | --- | --- | --- | --- | --- | --- | --- |
|  |  | DRS | | | | | | | | Test Value | Df | P-Value |
|  |  | Mild Stress | | Moderate Stress | | High Stress | | Severe Stress | |  |  |  |
| Sex | Male | 160 | 38% | 154 | 37% | 73 | 18% | 30 | 7% | 2.904a | 3 | 0.407 |
|  | Female | 276 | 35% | 295 | 37% | 168 | 21% | 61 | 8% |  |  |  |
| Does He / She Smoke? | Doesn’t Smoke | 384 | 36% | 398 | 37% | 202 | 19% | 83 | 8% | 4.934a | 3 | 0.177 |
|  | Smokes | 52 | 35% | 51 | 34% | 38 | 26% | 7 | 5% |  |  |  |
| Is Any of Your Parents a doctor? | Yes | 59 | 38% | 57 | 37% | 30 | 19% | 8 | 5% | 1.543a | 3 | 0.672 |
|  | No | 377 | 35% | 392 | 37% | 211 | 20% | 83 | 8% |  |  |  |
| Mother's Level of Education | Primary | 47 | 34% | 56 | 41% | 25 | 18% | 9 | 7% | 3.722a | 9 | 0.929 |
|  | Secondary | 96 | 34% | 108 | 38% | 55 | 19% | 25 | 9% |  |  |  |
|  | College | 214 | 37% | 204 | 35% | 122 | 21% | 41 | 7% |  |  |  |
|  | Associate Degree | 79 | 37% | 81 | 38% | 39 | 18% | 16 | 7% |  |  |  |
| Father’s Level of Education | Primary | 43 | 32% | 62 | 46% | 23 | 17% | 8 | 6% | 8.959a | 9 | 0.441 |
|  | Secondary | 87 | 35% | 88 | 35% | 59 | 23% | 18 | 7% |  |  |  |
|  | College | 243 | 36% | 249 | 37% | 130 | 19% | 54 | 8% |  |  |  |
|  | Associate Degree | 63 | 41% | 50 | 33% | 29 | 19% | 11 | 7% |  |  |  |
| Marital Status | Lonely | 430 | 36% | 436 | 36% | 238 | 20% | 91 | 8% | 5.555a | 3 | 0.135 |
|  | Married | 6 | 27% | 13 | 59% | 3 | 14% | 0 | 0% |  |  |  |
| Study Year | Second | 166 | 40% | 153 | 37% | 78 | 19% | 21 | 5% | 21.180a | 12 | 0.048 |
|  | Third | 77 | 30% | 106 | 41% | 50 | 19% | 25 | 10% |  |  |  |
|  | Fourth | 92 | 32% | 99 | 35% | 65 | 23% | 29 | 10% |  |  |  |
|  | Fifth | 89 | 41% | 72 | 33% | 40 | 19% | 14 | 7% |  |  |  |
|  | Sixth | 12 | 29% | 19 | 46% | 8 | 20% | 2 | 5% |  |  |  |
| Medical College | Medicine | 190 | 37% | 181 | 35% | 101 | 20% | 39 | 8% | 4.009a | 6 | 0.676 |
|  | Dentistry | 124 | 37% | 131 | 39% | 63 | 19% | 20 | 6% |  |  |  |
|  | Pharmacy | 122 | 33% | 137 | 37% | 77 | 21% | 32 | 9% |  |  |  |
| Grades of Last Semester | <70 | 41 | 30% | 47 | 34% | 32 | 23% | 18 | 13% | 16.735a | 9 | 0.053 |
|  | 70-79.99 | 152 | 34% | 165 | 37% | 96 | 21% | 35 | 8% |  |  |  |
|  | 80-89.99 | 202 | 37% | 202 | 37% | 102 | 19% | 33 | 6% |  |  |  |
|  | >=90 | 41 | 45% | 35 | 38% | 11 | 12% | 5 | 5% |  |  |  |
| Had Any Volunteer Experience | Yes | 189 | 35% | 218 | 40% | 97 | 18% | 40 | 7% | 4.970a | 3 | 0.174 |
|  | No | 247 | 37% | 231 | 34% | 144 | 21% | 51 | 8% |  |  |  |
| Place of Residence | City | 276 | 37% | 278 | 37% | 143 | 19% | 51 | 7% | 2.250a | 3 | 0.522 |
|  | Countryside | 160 | 34% | 171 | 36% | 98 | 21% | 40 | 9% |  |  |  |
|  | Inside Damascus | 279 | 35% | 299 | 38% | 154 | 19% | 61 | 8% | .977a | 3 | 0.807 |
|  | Outside Damascus | 157 | 37% | 150 | 35% | 87 | 21% | 30 | 7% |  |  |  |
| Sharing Residence with: | Parents | 363 | 35% | 368 | 36% | 210 | 21% | 82 | 8% | 11.106a | 9 | 0.268 |
|  | College | 37 | 40% | 36 | 39% | 15 | 16% | 4 | 4% |  |  |  |
|  | Rented | 26 | 37% | 27 | 38% | 14 | 20% | 4 | 6% |  |  |  |
|  | Relative | 10 | 32% | 18 | 58% | 2 | 6% | 1 | 3% |  |  |  |
| Economic Status | Low | 15 | 28% | 24 | 44% | 7 | 13% | 8 | 15% | 24.389a | 9 | 0.004 |
|  | Middle | 124 | 30% | 153 | 38% | 93 | 23% | 37 | 9% |  |  |  |
|  | Good | 267 | 39% | 238 | 35% | 126 | 19% | 46 | 7% |  |  |  |
|  | Perfect | 30 | 38% | 34 | 43% | 15 | 19% | 0 | 0% |  |  |  |
| Income | Parent | 385 | 36% | 384 | 36% | 217 | 20% | 75 | 7% | 18.304a | 12 | 0.107 |
|  | Private | 25 | 25% | 45 | 45% | 20 | 20% | 10 | 10% |  |  |  |
|  | Other | 10 | 43% | 10 | 43% | 2 | 9% | 1 | 4% |  |  |  |
|  | Both | 15 | 50% | 9 | 30% | 2 | 7% | 4 | 13% |  |  |  |

| Table-11 Cross-Tabulation of Group Activities Related Stressors Subscale of MSSQ with Demographics | | | | | | | | | | | | |
| --- | --- | --- | --- | --- | --- | --- | --- | --- | --- | --- | --- | --- |
|  |  | GARS | | | | | | | | Test Value | Df | P-Value |
|  |  | Mild Stress | | Moderate Stress | | High Stress | | Severe Stress | |  |  |  |
| Sex | Male | 114 | 27% | 179 | 43% | 98 | 24% | 26 | 6% | 20.128a | 3 | <.001 |
|  | Female | 154 | 19% | 315 | 39% | 244 | 31% | 87 | 11% |  |  |  |
| Does He / She Smoke? | Doesn’t Smoke | 223 | 21% | 436 | 41% | 303 | 28% | 105 | 10% | 8.577a | 3 | 0.035 |
|  | Smokes | 44 | 30% | 58 | 39% | 39 | 26% | 7 | 5% |  |  |  |
| Is Any of Your Parents a doctor? | Yes | 35 | 23% | 54 | 35% | 50 | 32% | 15 | 10% | 2.602a | 3 | 0.457 |
|  | No | 233 | 22% | 440 | 41% | 292 | 27% | 98 | 9% |  |  |  |
| Mother's Level of Education | Primary | 25 | 18% | 65 | 47% | 31 | 23% | 16 | 12% | 18.916a | 9 | 0.026 |
|  | Secondary | 53 | 19% | 128 | 45% | 70 | 25% | 33 | 12% |  |  |  |
|  | College | 136 | 23% | 225 | 39% | 169 | 29% | 51 | 9% |  |  |  |
|  | Associate Degree | 54 | 25% | 76 | 35% | 72 | 33% | 13 | 6% |  |  |  |
| Father’s Level of Education | Primary | 26 | 19% | 61 | 45% | 39 | 29% | 10 | 7% | 7.289a | 9 | 0.607 |
|  | Secondary | 53 | 21% | 109 | 43% | 66 | 26% | 24 | 10% |  |  |  |
|  | College | 152 | 22% | 256 | 38% | 202 | 30% | 66 | 10% |  |  |  |
|  | Associate Degree | 37 | 24% | 68 | 44% | 35 | 23% | 13 | 8% |  |  |  |
| Marital Status | Lonely | 261 | 22% | 483 | 40% | 338 | 28% | 113 | 9% | 4.330a | 3 | 0.228 |
|  | Married | 7 | 32% | 11 | 50% | 4 | 18% | 0 | 0% |  |  |  |
| Study Year | Second | 99 | 24% | 179 | 43% | 104 | 25% | 36 | 9% | 18.341a | 12 | 0.106 |
|  | Third | 48 | 19% | 104 | 40% | 81 | 31% | 25 | 10% |  |  |  |
|  | Fourth | 67 | 24% | 109 | 38% | 80 | 28% | 29 | 10% |  |  |  |
|  | Fifth | 50 | 23% | 86 | 40% | 65 | 30% | 14 | 7% |  |  |  |
|  | Sixth | 4 | 10% | 16 | 39% | 12 | 29% | 9 | 22% |  |  |  |
| Medical College | Medicine | 106 | 21% | 180 | 35% | 158 | 31% | 67 | 13% | 29.078a | 6 | <.001 |
|  | Dentistry | 69 | 20% | 150 | 44% | 89 | 26% | 30 | 9% |  |  |  |
|  | Pharmacy | 93 | 25% | 164 | 45% | 95 | 26% | 16 | 4% |  |  |  |
| Grades of Last Semester | <70 | 36 | 26% | 47 | 34% | 40 | 29% | 15 | 11% | 15.665a | 9 | 0.074 |
|  | 70-79.99 | 93 | 21% | 191 | 43% | 132 | 29% | 32 | 7% |  |  |  |
|  | 80-89.99 | 111 | 21% | 227 | 42% | 141 | 26% | 60 | 11% |  |  |  |
|  | >=90 | 28 | 30% | 29 | 32% | 29 | 32% | 6 | 7% |  |  |  |
| Had Any Volunteer Experience | Yes | 117 | 22% | 237 | 44% | 144 | 26% | 46 | 8% | 3.922a | 3 | 0.27 |
|  | No | 151 | 22% | 257 | 38% | 198 | 29% | 67 | 10% |  |  |  |
| Place of Residence | City | 179 | 24% | 292 | 39% | 204 | 27% | 73 | 10% | 5.312a | 3 | 0.15 |
|  | Countryside | 89 | 19% | 202 | 43% | 138 | 29% | 40 | 9% |  |  |  |
|  | Inside Damascus | 172 | 22% | 320 | 40% | 228 | 29% | 73 | 9% | .503a | 3 | 0.918 |
|  | Outside Damascus | 96 | 23% | 174 | 41% | 114 | 27% | 40 | 9% |  |  |  |
| Sharing Residence with: | Parents | 230 | 22% | 414 | 40% | 281 | 27% | 98 | 10% | 11.018a | 9 | 0.274 |
|  | College | 20 | 22% | 32 | 35% | 29 | 32% | 11 | 12% |  |  |  |
|  | Rented | 12 | 17% | 36 | 51% | 19 | 27% | 4 | 6% |  |  |  |
|  | Relative | 6 | 19% | 12 | 39% | 13 | 42% | 0 | 0% |  |  |  |
| Economic Status | Low | 15 | 28% | 15 | 28% | 15 | 28% | 9 | 17% | 20.966a | 9 | 0.013 |
|  | Middle | 77 | 19% | 166 | 41% | 126 | 31% | 38 | 9% |  |  |  |
|  | Good | 153 | 23% | 288 | 43% | 172 | 25% | 64 | 9% |  |  |  |
|  | Perfect | 23 | 29% | 25 | 32% | 29 | 37% | 2 | 3% |  |  |  |
| Income | Parent | 229 | 22% | 427 | 40% | 302 | 28% | 103 | 10% | 4.513a | 12 | 0.972 |
|  | Private | 24 | 24% | 45 | 45% | 25 | 25% | 6 | 6% |  |  |  |
|  | Other | 7 | 30% | 9 | 39% | 6 | 26% | 1 | 4% |  |  |  |
|  | Both | 7 | 23% | 12 | 40% | 8 | 27% | 3 | 10% |  |  |  |

| Table-12 Cross-Tabulation of PHQ4 Scale with Demographics | | | | | | | | | | |
| --- | --- | --- | --- | --- | --- | --- | --- | --- | --- | --- |
|  | | PHQ4 | | | | | | Test Value | Df | P Value |
|  |  | Normal | | Milld | | Moderate | |  |  |  |
| Sex | Male | 80 | 24% | 142 | 42% | 117 | 35% | 49.252a | 3 | <0.001 |
|  | Female | 63 | 11% | 235 | 43% | 250 | 46% |  |  |  |
| Does He / She Smoke? | Doesn’t Smoke | 117 | 15% | 328 | 43% | 326 | 42% | 6.706a | 3 | 0.082 |
|  | Smokes | 26 | 23% | 48 | 42% | 41 | 36% |  |  |  |
| Is Any of Your Parents a doctor? | Yes | 25 | 22% | 48 | 42% | 40 | 35% | 4.057a | 3 | 0.255 |
|  | No | 118 | 15% | 329 | 43% | 327 | 42% |  |  |  |
| Mother's Level of Education | Primary | 15 | 16% | 48 | 51% | 31 | 33% | 16.159a | 9 | 0.064 |
|  | Secondary | 30 | 14% | 80 | 38% | 103 | 48% |  |  |  |
|  | College | 75 | 18% | 190 | 45% | 156 | 37% |  |  |  |
|  | Associate Degree | 23 | 14% | 59 | 37% | 77 | 48% |  |  |  |
| Father’s Level of Education | Primary | 12 | 13% | 38 | 41% | 43 | 46% | 11.449a | 9 | 0.246 |
|  | Secondary | 23 | 13% | 77 | 42% | 84 | 46% |  |  |  |
|  | College | 90 | 18% | 210 | 43% | 188 | 39% |  |  |  |
|  | Associate Degree | 18 | 15% | 52 | 43% | 52 | 43% |  |  |  |
| Marital Status | Lonely | 139 | 16% | 368 | 42% | 363 | 42% | 2.727a | 3 | 0.436 |
|  | Married | 4 | 24% | 9 | 53% | 4 | 24% |  |  |  |
| Study Year | Second | 54 | 18% | 133 | 43% | 121 | 39% | 8.496a | 12 | 0.745 |
|  | Third | 24 | 13% | 75 | 41% | 86 | 46% |  |  |  |
|  | Fourth | 35 | 17% | 83 | 41% | 84 | 42% |  |  |  |
|  | Fifth | 26 | 16% | 69 | 42% | 68 | 42% |  |  |  |
|  | Sixth | 4 | 14% | 17 | 59% | 8 | 28% |  |  |  |
| Medical College | Medicine | 64 | 18% | 156 | 43% | 144 | 40% | 11.879a | 6 | 0.065 |
|  | Dentistry | 40 | 17% | 91 | 39% | 104 | 44% |  |  |  |
|  | Pharmacy | 39 | 14% | 130 | 45% | 119 | 41% |  |  |  |
| Grades of Last Semester | <70 | 12 | 12% | 38 | 39% | 48 | 49% | 13.367a | 9 | 0.147 |
|  | 70-79.99 | 45 | 14% | 149 | 45% | 135 | 41% |  |  |  |
|  | 80-89.99 | 72 | 19% | 153 | 40% | 162 | 42% |  |  |  |
|  | >=90 | 14 | 19% | 37 | 51% | 22 | 30% |  |  |  |
| Had Any Volunteer Experience | Yes | 65 | 16% | 180 | 45% | 156 | 39% | 2.411a | 3 | 0.492 |
|  | No | 78 | 16% | 197 | 41% | 211 | 43% |  |  |  |
| Place of Residence | City | 85 | 16% | 230 | 42% | 229 | 42% | .433a | 3 | 0.933 |
|  | Countryside | 58 | 17% | 147 | 43% | 138 | 40% |  |  |  |
|  | Inside Damascus | 82 | 14% | 240 | 42% | 250 | 44% | 6.115a | 3 | 0.106 |
|  | Outside Damascus | 61 | 19% | 137 | 43% | 117 | 37% |  |  |  |
| Sharing Residence with: | Parents | 112 | 15% | 316 | 43% | 312 | 42% | 5.064a | 9 | 0.829 |
|  | College | 15 | 22% | 30 | 43% | 24 | 35% |  |  |  |
|  | Rented | 11 | 20% | 22 | 40% | 22 | 40% |  |  |  |
|  | Relative | 5 | 22% | 9 | 39% | 9 | 39% |  |  |  |
| Economic Status | Low | 6 | 19% | 12 | 38% | 14 | 44% | 11.142a | 9 | 0.266 |
|  | Middle | 46 | 16% | 125 | 43% | 119 | 41% |  |  |  |
|  | Good | 80 | 16% | 209 | 41% | 216 | 43% |  |  |  |
|  | Perfect | 11 | 18% | 31 | 52% | 18 | 30% |  |  |  |
| Income | Parent | 124 | 16% | 334 | 43% | 316 | 41% | 5.076a | 12 | 0.955 |
|  | Private | 13 | 18% | 28 | 38% | 32 | 44% |  |  |  |
|  | Other | 2 | 11% | 7 | 39% | 9 | 50% |  |  |  |
|  | Both | 4 | 19% | 8 | 38% | 9 | 43% |  |  |  |

| Table-13 Cross-Tabulation of Anxiety Subscale of PHQ-4 with Demographics | | | | | | | | |
| --- | --- | --- | --- | --- | --- | --- | --- | --- |
|  | | Phq-4 Anxiety | | | | Test Value | Df | P-Value |
|  |  | No Anxiety | | Anxiety | |  |  |  |
| Sex | Male | 223 | 53% | 194 | 47% | 45.454a | 1 | <0.001 |
|  | Female | 268 | 34% | 532 | 67% |  |  |  |
| Does He / She Smoke? | Doesn’t Smoke | 421 | 39% | 646 | 61% | 2.773a | 1 | 0.096 |
|  | Smokes | 69 | 47% | 79 | 53% |  |  |  |
| Is Any of Your Parents a doctor? | Yes | 72 | 47% | 82 | 53% | 3.008a | 1 | 0.083 |
|  | No | 419 | 39% | 644 | 61% |  |  |  |
| Mother's Level of Education | Primary | 54 | 39% | 83 | 61% | 5.106a | 3 | 0.164 |
|  | Secondary | 101 | 36% | 183 | 64% |  |  |  |
|  | College | 252 | 43% | 329 | 57% |  |  |  |
|  | Associate Degree | 84 | 39% | 131 | 61% |  |  |  |
| Father’s Level of Education | Primary | 49 | 36% | 87 | 64% | 2.991a | 3 | 0.393 |
|  | Secondary | 94 | 37% | 158 | 63% |  |  |  |
|  | College | 285 | 42% | 391 | 58% |  |  |  |
|  | Associate Degree | 63 | 41% | 90 | 59% |  |  |  |
| Marital Status | Lonely | 480 | 40% | 715 | 60% | .868a | 1 | 0.352 |
|  | Married | 11 | 50% | 11 | 50% |  |  |  |
| Study Year | Second | 189 | 45% | 229 | 55% | 6.454a | 4 | 0.168 |
|  | Third | 99 | 38% | 159 | 62% |  |  |  |
|  | Fourth | 105 | 37% | 180 | 63% |  |  |  |
|  | Fifth | 82 | 38% | 133 | 62% |  |  |  |
|  | Sixth | 16 | 39% | 25 | 61% |  |  |  |
| Medical College | Medicine | 213 | 42% | 298 | 58% | 7.546a | 2 | 0.023 |
|  | Dentistry | 116 | 34% | 222 | 66% |  |  |  |
|  | Pharmacy | 162 | 44% | 206 | 56% |  |  |  |
| Grades of Last Semester | <70 | 52 | 38% | 86 | 62% | 8.475a | 3 | 0.037 |
|  | 70-79.99 | 173 | 39% | 275 | 61% |  |  |  |
|  | 80-89.99 | 216 | 40% | 323 | 60% |  |  |  |
|  | >=90 | 50 | 54% | 42 | 46% |  |  |  |
| Had Any Volunteer Experience | Yes | 218 | 40% | 326 | 60% | .030a | 1 | 0.862 |
|  | No | 273 | 41% | 400 | 59% |  |  |  |
| Place of Residence | City | 302 | 40% | 446 | 60% | .001a | 1 | 0.979 |
|  | Countryside | 189 | 40% | 280 | 60% |  |  |  |
|  | Inside Damascus | 301 | 38% | 492 | 62% | 5.393a | 1 | 0.02 |
|  | Outside Damascus | 190 | 45% | 234 | 55% |  |  |  |
| Sharing Residence with: | Parents | 403 | 39% | 620 | 61% | 2.737a | 3 | 0.434 |
|  | College | 40 | 43% | 52 | 57% |  |  |  |
|  | Rented | 34 | 48% | 37 | 52% |  |  |  |
|  | Relative | 14 | 45% | 17 | 55% |  |  |  |
| Economic Status | Low | 19 | 35% | 35 | 65% | 3.648a | 3 | 0.302 |
|  | Middle | 155 | 38% | 252 | 62% |  |  |  |
|  | Good | 279 | 41% | 398 | 59% |  |  |  |
|  | Perfect | 38 | 48% | 41 | 52% |  |  |  |
| Income | Parent | 431 | 41% | 630 | 59% | 3.439a | 4 | 0.487 |
|  | Private | 36 | 36% | 64 | 64% |  |  |  |
|  | Other | 10 | 43% | 13 | 57% |  |  |  |
|  | Both | 14 | 47% | 16 | 53% |  |  |  |

| Table-14 Cross-Tabulation of Depression Subscale of PHQ-4 with Demographics | | | | | | | | |
| --- | --- | --- | --- | --- | --- | --- | --- | --- |
|  | | Phq-4 Depression | | | | Test Value | Df | P-Value |
|  |  | No Anxiety | | Anxiety | |  |  |  |
| Sex | Male | 197 | 47% | 220 | 53% | 10.768a | 1 | 0.001 |
|  | Female | 300 | 38% | 500 | 63% |  |  |  |
| Does He / She Smoke? | Doesn’t Smoke | 426 | 40% | 641 | 60% | 3.483a | 1 | 0.062 |
|  | Smokes | 69 | 47% | 79 | 53% |  |  |  |
| Is Any of Your Parents a doctor? | Yes | 67 | 44% | 87 | 56% | .520a | 1 | 0.471 |
|  | No | 430 | 40% | 633 | 60% |  |  |  |
| Mother's Level of Education | Primary | 56 | 41% | 81 | 59% | .119a | 3 | 0.99 |
|  | Secondary | 115 | 40% | 169 | 60% |  |  |  |
|  | College | 236 | 41% | 345 | 59% |  |  |  |
|  | Associate Degree | 90 | 42% | 125 | 58% |  |  |  |
| Father’s Level of Education | Primary | 51 | 38% | 85 | 63% | 2.841a | 3 | 0.417 |
|  | Secondary | 99 | 39% | 153 | 61% |  |  |  |
|  | College | 276 | 41% | 400 | 59% |  |  |  |
|  | Associate Degree | 71 | 46% | 82 | 54% |  |  |  |
| Marital Status | Lonely | 481 | 40% | 714 | 60% | 9.430a | 1 | 0.002 |
|  | Married | 16 | 73% | 6 | 27% |  |  |  |
| Study Year | Second | 174 | 42% | 244 | 58% | 8.268a | 4 | 0.082 |
|  | Third | 86 | 33% | 172 | 67% |  |  |  |
|  | Fourth | 123 | 43% | 162 | 57% |  |  |  |
|  | Fifth | 95 | 44% | 120 | 56% |  |  |  |
|  | Sixth | 19 | 46% | 22 | 54% |  |  |  |
| Medical College | Medicine | 208 | 41% | 303 | 59% | 1.279a | 2 | 0.528 |
|  | Dentistry | 131 | 39% | 207 | 61% |  |  |  |
|  | Pharmacy | 158 | 43% | 210 | 57% |  |  |  |
| Grades of Last Semester | <70 | 48 | 35% | 90 | 65% | 6.302a | 3 | 0.098 |
|  | 70-79.99 | 179 | 40% | 269 | 60% |  |  |  |
|  | 80-89.99 | 223 | 41% | 316 | 59% |  |  |  |
|  | >=90 | 47 | 51% | 45 | 49% |  |  |  |
| Had Any Volunteer Experience | Yes | 230 | 42% | 314 | 58% | .846a | 1 | 0.358 |
|  | No | 267 | 40% | 406 | 60% |  |  |  |
| Place of Residence | City | 304 | 41% | 444 | 59% | .031a | 1 | 0.86 |
|  | Countryside | 193 | 41% | 276 | 59% |  |  |  |
|  | Inside Damascus | 313 | 39% | 480 | 61% | 1.762a | 1 | 0.184 |
|  | Outside Damascus | 184 | 43% | 240 | 57% |  |  |  |
| Sharing Residence with: | Parents | 409 | 40% | 614 | 60% | 3.466a | 3 | 0.325 |
|  | College | 43 | 47% | 49 | 53% |  |  |  |
|  | Rented | 34 | 48% | 37 | 52% |  |  |  |
|  | Relative | 11 | 35% | 20 | 65% |  |  |  |
| Economic Status | Low | 14 | 26% | 40 | 74% | 7.474a | 3 | 0.058 |
|  | Middle | 164 | 40% | 243 | 60% |  |  |  |
|  | Good | 280 | 41% | 397 | 59% |  |  |  |
|  | Perfect | 39 | 49% | 40 | 51% |  |  |  |
| Income | Parent | 435 | 41% | 626 | 59% | 2.904a | 4 | 0.574 |
|  | Private | 42 | 42% | 58 | 58% |  |  |  |
|  | Other | 10 | 43% | 13 | 57% |  |  |  |
|  | Both | 10 | 33% | 20 | 67% |  |  |  |
